# Hepatic ARID3A facilitates liver cancer malignancy by cooperating with CEP131 to regulate an embryonic stem cell-like gene signature

**DOI:** 10.1038/s41419-022-05187-9

**Published:** 2022-08-25

**Authors:** Mengting Shen, Shengli Li, Yiming Zhao, Yizhe Liu, Zhen Liu, Lin Huan, Yejun Qiao, Lu Wang, Leng Han, Zhiao Chen, Xianghuo He

**Affiliations:** 1 Fudan University Shanghai Cancer Center and Institutes of Biomedical Sciences, Shanghai Medical College, Fudan University, Shanghai, 200032 China; 2 Precision Research Center for Refractory Diseases, Institute for Clinical Research, Shanghai General Hospital, Shanghai Jiao Tong University School of Medicine, Shanghai, 201620 China; 3 Department of Hepatic Surgery, Fudan University Shanghai Cancer Center, Fudan University, Shanghai, 200032 China; 4 Center for Epigenetics and Disease Prevention, Institute of Biosciences and Technology, Texas A&M University, Houston, TX 77030 USA; 5 Key Laboratory of Breast Cancer in Shanghai, Fudan University Shanghai Cancer Center, Fudan University, Shanghai, 200032 China; 6 Shanghai Key Laboratory of Radiation Oncology, Fudan University Shanghai Cancer Center, Fudan University, Shanghai, 200032 China

**Keywords:** Metastasis, Liver cancer, Oncogenes

## Abstract

Liver cancer stemness refers to the stem cell-like phenotype of hepatocarcinoma cells and is closely related to a high degree of tumour malignancy. Here, we identified AT-rich interacting domain 3A (ARID3A) as one of the most upregulated stemness-related transcription factors in liver cancer by an in vitro functional screen. ARID3A can promote liver cancer cell viability and metastasis both in vitro and in vivo. Mechanistically, ARID3A interacts with CEP131 and transcriptionally activates KDM3A by co-occupying its promoter element, further upregulating the expression of downstream embryonic stem (ES) signature genes via demethylation of H3K9me2. ARID3A and CEP131 promote an ES cell gene signature through activation of KDM3A and contribute to the poor prognosis of liver cancer patients. Collectively, these results provide evidence highlighting a transcription-dependent mechanism of ARID3A in stemness regulation in liver cancer. The ARID3A/CEP131-KDM3A regulatory circuit could serve as a prognostic indicator and potential therapeutic target for liver cancer.

## Introduction

Primary liver cancers, including hepatocellular carcinoma (HCC) and intrahepatic cholangiocarcinoma as well as other rare types, have the sixth highest incidence and rank third in cancer-related deaths worldwide [1]. Despite continuous clinical advances in therapies over the past decades, resistance to chemotherapy and high rates of recurrence have led to the poor prognosis of liver cancer [2]. Liver cancer cell stemness is vital components that influence liver cancer malignancy [3, 4].

Each of the iPSC (induced pluripotent stem cell) reprogramming factors has established roles in oncogenesis in many tumours. The abnormally high expression of totipotent transcription factors (TFs) in liver cancer patients can endow tumours with increased aggressiveness, which is associated with poorer clinical prognosis [5–7]. These results reveal the role of pluripotency-related transcription factors in liver cancer initiation and development. However, in the current research on liver cancer, whether there are other transcription factors-in addition to the classical pluripotency-related stemness transcription factors-that act as stemness transcription factors in liver cancer has not been reported in detail.

Given the importance of dysregulated transcriptional programs in liver cancer, we analysed expression profiles of paired liver cancer samples followed by in vitro knockout screen, and we found that AT-rich interacting domain 3A (ARID3A) is a potent oncogenic TF and is highly related to embryonic stem (ES) cell-like gene expression signature (ES signature). ARID3A has been reported to participate in a variety of biological processes, such as gene expression, stem cell fate decisions, chromatin accessibility, and cell cycle regulation [8, 9]. However, deficiency of ARID3A induces the expression of a number of pluripotency-associated gene products in mouse models and alterations in colony morphology in human cells, indicating that ARID3A probably has two roles in regulating cell stemness [10]. In liver cancer, integrated analysis of gene expression profiles of HCC led to the construction of a transcriptional regulatory network that contained most downstream DEGs (differentially expressed genes), including ARID3A [11]. However, the molecular mechanisms underlying the oncogenic role of ARID3A in liver cancer remain largely unexplored.

Here, we found that ARID3A is upregulated in liver cancer. ARID3A regulates the viability of liver cancer cells and influences metastasis both in vitro and in vivo. Interestingly, ARID3A can regulate the expression of KDM3A, thereby driving the expression of ES signature genes through H3 lysine 9 dimethylation (H3K9me2) in liver cancer cells. Moreover, coimmunoprecipitation (Co-IP) and liquid chromatography-mass spectrometry (LC-MS) analysis revealed CEP131 as a binding partner of ARID3A. ARID3A can cooperate with CEP131 to transcriptionally activate KDM3A and promote expression of ES signature genes, suggesting that ARID3A and CEP131 are potential targets for the development of new anticancer therapeutics for liver cancer.

## Results

### High expression of ARID3A correlates with ES cell-like gene expression signature and poor outcomes in liver cancer patients

**Fig. 1:**
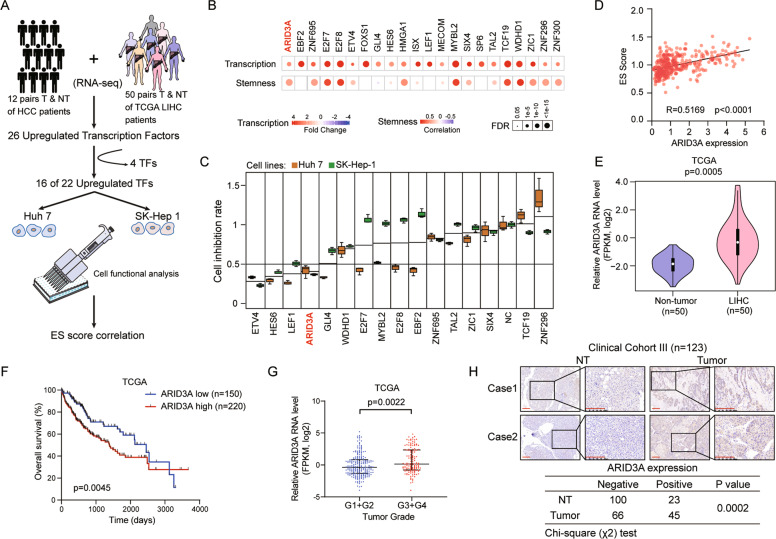
High expression of ARID3A correlates with poor outcomes and cancer stemness in liver cancer patients. **A** Schematic diagram of the protocol used to search for candidate oncogenic TFs in liver cancer. **B** Upregulation patterns and correlation levels with ES scores of 22 selected TFs in patients in the TCGA-LIHC cohort. The colour intensity indicates the fold change or correlation coefficient, and the point size indicates the statistical significance at a given FDR. Two-tailed Student’s *t-*test or Spearman’s rank correlation analysis and followed by FDR adjustment for *p*-value. **C** The cell growth inhibition rate after transfection of siRNA into Huh7 and SK-Hep-1 cells was determined on Day 5 using a CCK-8 assay. The values are expressed as the means ± SEMs, *n* = 3. biologically independent experiments. **D** The correlation between the ES scores of individual patient samples and ARID3A expression was determined in the TCGA-LIHC dataset. *n* = 374, Spearman’s rank correlation analysis. **E** ARID3A expression was increased in tumour tissues in 50 paired samples in the TCGA-LIHC cohort, two-tailed Student’s *t*-test. **F** Kaplan–Meier analysis of overall survival for liver cancer patients in the TCGA-LIHC cohort (*n* = 370), Log-rank test. **G** The expression of ARID3A in LIHC patients with different tumour grades in the TCGA cohort. *n* = 232 for G1 + 2 and *n* = 134 for G3 + 4, two-tailed Student’s *t*-test. The values are expressed as the medians with interquartile ranges. **H** IHC analysis of ARID3A in tumour and adjacent nontumour (NT) tissues (scale bar, 200 μm). *n* = 111 tumour tissues and *n* = 123 NT tissues, chi-square test.

We first performed integrated analysis of the transcriptomic data from 50 paired HCC tumour and adjacent nontumour (NT) liver tissues in the TCGA-LIHC (The Cancer Genome Atlas-HCC) dataset and our twelve paired Chinese liver cancer samples (GSE101432) (Fig. 1A). By taking the intersection of the two cohorts, we identified 26 upregulated TFs with twofold upregulation in tumour tissues compared with NT liver tissues, although four of them (E2F1, FOXM1, LIN28B, and MYCN) were excluded for overrepresentation. Next, the remaining 22 TFs were used to establish ES cell-like gene expression signature scores (ES scores) based on TCGA gene expression data and ranked by their gene expression level in 7 liver cancer cell lines (Figs. 1B and S1A); 16 of them were then selected for functional screening using the siRNA approach in two liver cancer cell lines [12]. We identified 4 TFs, ETV4, HES6, LEF1 and ARID3A, associated with a significantly decreased cell proliferation rate following transfection of siRNAs against each TF in Huh7 and SK-Hep-1 cells (with 0.5 as the cut-off, Fig. 1C). Notably, ARID3A showed the highest correlation (in terms of the correlation coefficient (*r*)) with stem cell characteristics (Fig. 1D), indicating that dysregulation of ARID3A may play a key role in cancer development and progression.

The mRNA level of ARID3A was notably upregulated in tumour tissues (Fig. 1E), and a high ARID3A level was markedly associated with poor overall survival in liver cancer patients (Fig. 1F). In addition, these results were confirmed in 101 paired liver cancer tissues in an independent cohort (cohort II) (Fig. S1B, C). Moreover, patients with a poor histologic grade, metastasis and tumour thrombus had higher expression of ARID3A (Fig. 1G and Table S1, S2). Furthermore, positive ARID3A expression was found in 45 of the 111 (40.5%) primary liver cancer samples and in 23 of the 123 (18.7%) adjacent NT tissues in cohort III (*P* = 0.0002) (Fig. 1H).

### ARID3A is essential for the properties of liver cancer cell stemness

**Fig. 2:**
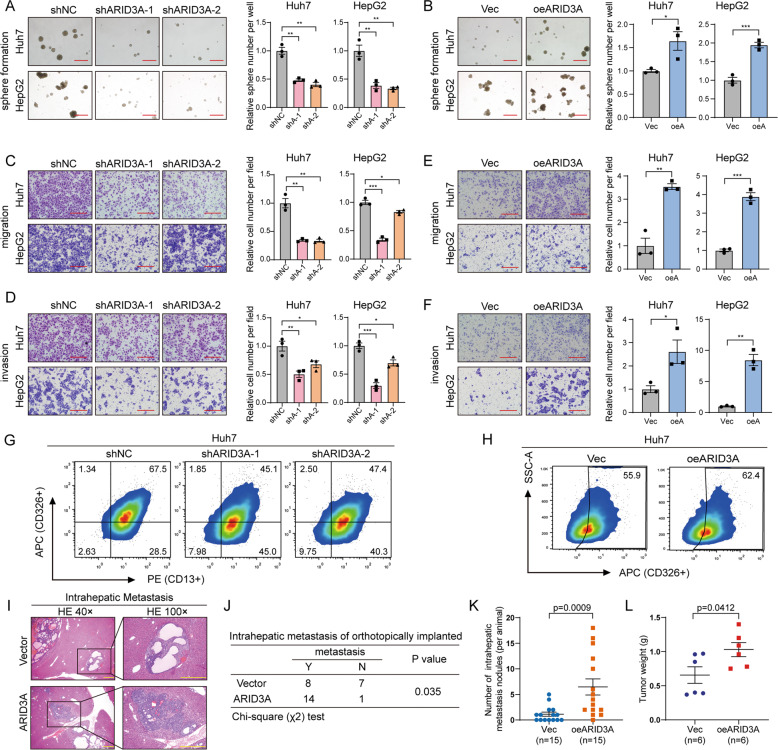
ARID3A promotes liver cancer cell tumoursphere formation, migration, invasion and metastasis. **A**, **B** Tumoursphere formation assays of Huh7 and HepG2 cells after ARID3A knockdown (shNC as the control) (**A**) and ARID3A overexpression (Vec as the control) (**B**). *n* = 3 biologically independent experiments, one-way ANOVA with Tukey’s multiple comparison test or two-tailed Student’s *t-*test (scale bar, 300 μm). **C**–**F** Transwell migration and invasion assays of Huh7 and HepG2 cells after ARID3A knockdown (shNC as the control) (**C**, **D**) and ARID3A overexpression (Vec as the control) (**E**, **F**). *n* = 3 biologically independent experiments, one-way ANOVA with Tukey’s multiple comparison test or two-tailed Student’s *t*-test (scale bar, 150 μm). **G**, **H** FACS analysis of the cell surface markers CD326 and CD13 in Huh7 cells after ARID3A knockdown (**G**) or ARID3A overexpression (**H**). The results shown are from a representative experiment repeated multiple times on different occasions. **I** Haematoxylin-eosin (H&E) staining of sections with metastatic nodules in mouse livers after transplantation of Huh7 cells stably overexpressing ARID3A and control cells (scale bar, 400 μm). **J** The prometastatic role of ARID3A was indicated in the mouse model. *n* = 15 mice, chi-square test. **K** The numbers of intrahepatic metastatic nodules were determined and analysed separately; Mann–Whitney *U*-test. **L** Tumour weights were measured in the ARID3A-overexpressing and control groups in the xenograft mouse model; Mann–Whitney *U*-test. The values are expressed as the means ± SEMs; *p < 0.05, **p < 0.01, and ***p < 0.001.

ARID3A is mainly expressed in reproductive and developmental tissues, such as the placenta, testis and bone marrow, while it is barely expressed in normal liver tissue, indicating that it may have roles in embryonic patterning and cell lineage gene regulation (Fig. S2A). Given that ARID3A expression is often increased in liver cancer and is highly related to stem cell characteristics, we first evaluated the effect of ARID3A on the tumoursphere formation capability. Knockdown of ARID3A dramatically reduced the tumoursphere formation capability, while overexpression of ARID3A significantly promoted cell sphere formation in Huh7 and HepG2 cells (Figs. 2A, B, and S2B). Interestingly, after ARID3A knockdown, the proliferation of liver cancer cells was significantly diminished (Fig. S2C). However, overexpression of ARID3A did not affect the proliferation of liver cancer cells (Fig. S2D). Moreover, the results of transwell assays, together with those of wound healing assays, revealed that a higher ARID3A level notably enhanced the migration and invasion capabilities of liver cancer cells. In addition, knockdown of ARID3A suppressed cell migration and invasion (Figs. 2C–F and S2E). Similarly, overexpression of ARID3A strongly promoted the migration and invasion of CLC2 and CLC7 cells derived from Chinese liver cancer patients (Fig. S2F, G). ARID3A also can promote the migration, invasion and tumoursphere formation capabilities of c-Myc-transformed HiHep cells (Fig. S2H, I), which indicated that ARID3A was involved in liver cancer progression [13]. As demonstrated by their enhancement of tumorigenicity, the liver cancer stemness markers CD326, CD133, CD44, ALDH1A1 and CD13 were found mostly downregulated in ARID3A knockdown cells and upregulated in ARID3A overexpressed cells (Fig. S2J, K). CD13 and CD326 (EpCAM) are well-established surface markers of cancer stemness in liver cancer [14, 15]. Interestingly, fluorescence-activated cell sorting (FACS) analysis showed that the proportions of CD13 + /CD326 + cells among Huh7 cells were decreased after ARID3A knockdown, whereas the proportion of CD326 + cells among Huh7 cells was increased after ARID3A overexpression (Fig. 2G, H).

To further explore the effect of ARID3A on liver cancer cell metastasis in vivo, Huh7 cells stably overexpressing ARID3A and control cells were transplanted into the livers of BALB/c nude mice (Fig. 2I). Importantly, the number of mice with intrahepatic metastasis and the numbers of metastatic nodules in the livers of individual mice were considerably increased in the ARID3A overexpression group compared with the vector control group (Fig. 2J, K). Interestingly, immunohistochemical (IHC) analysis of the xenografts showed that the ARID3A-positive cells were mainly located at the periphery of the tumour, indicating that ARID3A is related to cell stemness regulation in vivo (Fig. S2L) [16, 17]. We also evaluated the effect of ARID3A on liver cancer cell growth in vivo. Cells with stable ARID3A-overexpression generated from Huh7 cells were subcutaneously injected into nude mice, and the weights of the tumours were found to be much higher in the ARID3A-overexpressing group than in the control group (Fig. 2L and Fig. S2M). Taken together, these data suggest that ARID3A promotes cancer cell viability and metastasis both in vitro and in vivo.

### ARID3A-mediated regulation of ES signature is characterized by a stem cell population maintenance pathway

**Fig. 3:**
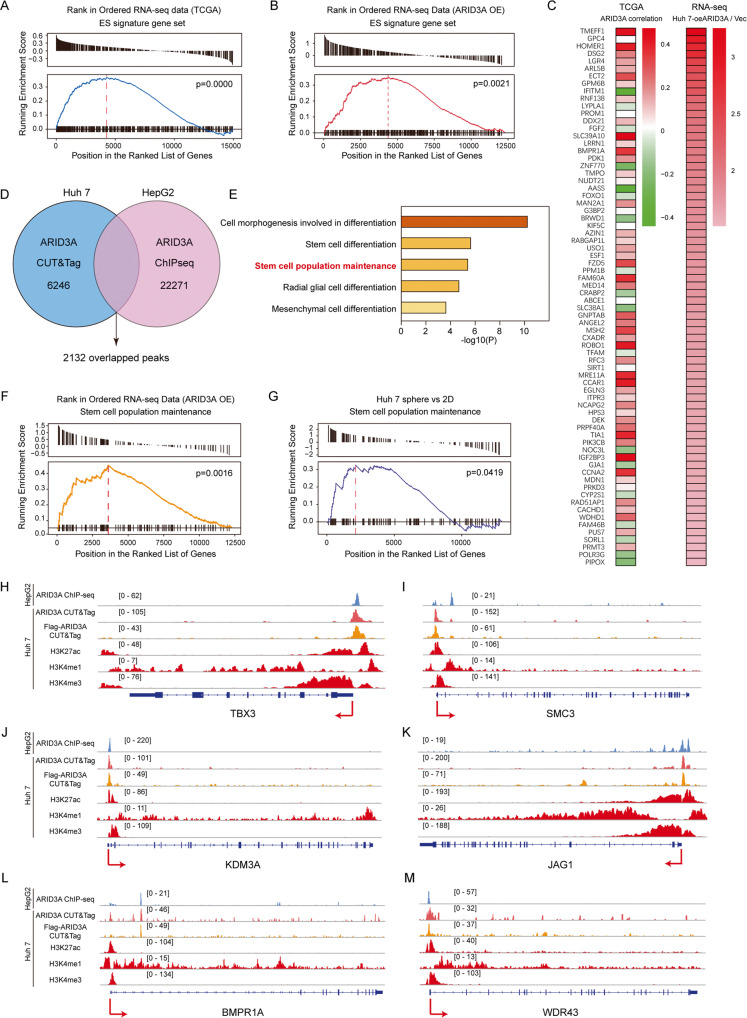
ARID3A regulates the expression of stem cell population maintenance genes. **A** Association between the enrichment of ES signature genes and ARID3A expression in HCC tumours from patients in the TCGA-LIHC cohort, as determined by GSEA. **B** GSEA of the ES signature gene set in RNA-seq data from Huh7 cells overexpressing ARID3A (vs. control vector cells). **C** Left: Correlation patterns between the expression of ARID3A and the subsets of ES signature genes. Right: mRNA expression levels of ES signature gene sets in ARID3A-overexpressing Huh7 cells. **D** Overlap of genomic loci corresponding to ARID3A CUT&Tag data in Huh7 cells and ARID3A ChIP-seq data in HepG2 cells (ENCODE: ENCSR000EDP). **E** GO term enrichment analysis of stemness-associated genes related to the overlapping loci in Fig. 3D. **F**, **G** GSEA of the stem cell population maintenance pathway in ARID3A-overexpressing Huh7 cells (vs. control, vector-overexpressing cells) (**F**) and Huh7 cell tumourspheres (vs. 2D cultured cells) (**G**). **H**–**M** Profiles of ARID3A, H3K27ac, H3K4me1 and H3K4me3 occupancy at the promoter regions of the indicated genes.

As mentioned above, gene set enrichment analysis (GSEA) showed that ARID3A-associated genes in patients in the TCGA-LIHC cohort were enriched in the ES signature (Fig. 3A). We then performed RNA sequencing (RNA-seq) to elucidate the underlying transcriptional programs affecting ARID3A activity. The genes that were most significantly upregulated in ARID3A-overexpressing Huh7 cells or downregulated in ARID3A knockdown Huh7 cells compared with control cells were enriched in the ES signature (Figs. 3B and S3A). We defined ES signature genes whose expression was increased by >1.5-fold by ARID3A overexpression as ARID3A-associated ES genes (Fig. 3C). To identify these genes that are directly regulated by ARID3A, we performed cleavage under targets and tagmentation (CUT&Tag) in Huh7 cells using an anti-ARID3A antibody. However, CUT&Tag revealed that only 10 of the 72 ARID3A-associated ES genes were bound directly by ARID3A in their promoters near transcription start sites (TSSs) (Fig. S3B), suggesting that ARID3A might not affect ES signature genes by directly regulating the transcriptional activity of these genes.

To explore how these genes are regulated by ARID3A, we focused on the overlapping downstream targets occupied by ARID3A in Huh7 cells and in HepG2 cells (Figs. 3D and S3C). Importantly, ARID3A-binding regions showed good correlations with H3K27ac signals, suggesting that transcriptional activation was associated with the binding of ARID3A (Fig. S3D). Genes associated with ARID3A-enriched regions genes were annotated, and Gene Ontology (GO) analysis showed that these genes were strongly enriched in the stem cell population maintenance pathway (Fig. 3E). Interestingly, we observed strong enrichment of the upregulated transcripts in the stem cell population maintenance pathway in ARID3A-overexpressing Huh7 cells compared with control Huh7 cells (Fig. 3F). In addition, this pathway was enriched in the tumourspheres compared with adherent cells (Fig. 3G). Figure 3H–M shows the ARID3A-bound regions at the loci of genes in the stem cell population maintenance pathway. These regions included numerous ARID3A-responsive genes with strong H3K27ac and H3K4me3 signals localized at the promoter regions, suggesting that transcriptional activation is associated with the binding of ARID3A.

### Identification of KDM3A as a key target for ARID3A-mediated regulation of ES signature genes

**Fig. 4:**
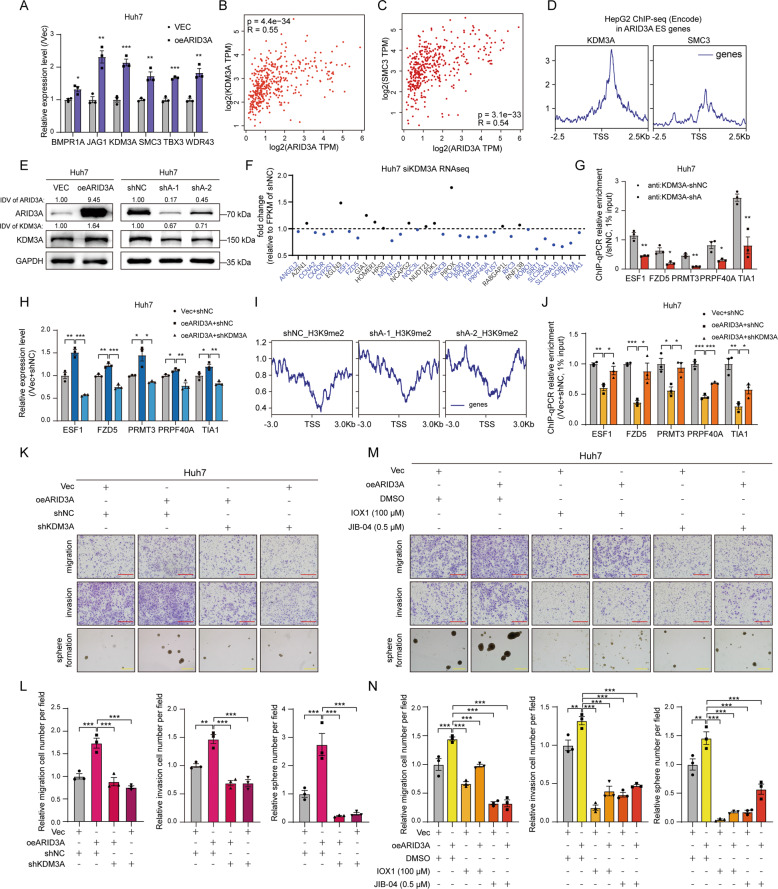
ARID3A activates ES signature genes expression through H3K9me2 demethylation by KDM3A. **A** The mRNA expression levels of stem cell population maintenance genes were determined by qPCR in Huh7 cells after ARID3A overexpression. *n* = 3 biologically independent experiments, two-tailed Student’s *t-*test. **B**, **C** Correlations between KDM3A (**B**) or SMC3 (**C**) expression and ARID3A expression in TCGA-LIHC (tumour and normal) were determined from the GEPIA database by Spearman’s rank correlation analysis. **D** Genomic occupancy profile of KDM3A (ENCODE: ENCSR387JKT) and SMC3 (ENCODE: ENCSR000EDW) in the TSS regions of the ARID3A-associated ES genes in HepG2 cell ChIP-seq data. **E** KDM3A protein expression levels were determined by western blot analysis in Huh7 cells after ARID3A overexpression or knockdown. The integrated density value (IDV) was calculated by ImageJ and normalized to the internal control. **F** Relative mRNA levels of KDM3A-related ARID3A-associated ES genes in KDM3A knockdown Huh7 cells (vs. siNC cells) were determined by RNA-seq. **G** The KDM3A occupancy levels in the promoters of representative ARID3A-associated ES signature genes were determined by ChIP-qPCR in ARID3A knockdown Huh7 cells (vs. shNC cells). *n* = 3 biologically independent experiments, two-tailed Student’s *t*-test. **H** The mRNA levels of representative ARID3A-associated ES genes in Huh7 cells treated as indicate. *n* = 3 biologically independent experiments, one-way ANOVA with Tukey’s multiple comparison test. **I** Genomic occupancy profile of H3K9me2 in the TSS regions of ARID3A-associated ES genes in ARID3A knockdown Huh7 cells (vs. shNC cells). **J** The H3K9me2 occupancy levels in the promoters of representative ARID3A-associated ES genes were determined by ChIP-qPCR after the indicated treatments. *n* = 3 biologically independent experiments, one-way ANOVA with Tukey’s multiple comparison test. **K**, **L** Transwell and tumoursphere formation assays were performed in Huh7 cells treated as indicated (red scale bar, 150 μm, yellow scale bar, 300 μm). *n* = 3 biologically independent experiments, one-way ANOVA with Tukey’s multiple comparison test. **M**, **N** Transwell and tumoursphere formation assays were performed in ARID3A-overexpressing Huh7 cells treated with the KDM3A inhibitors IOX1 and JIB-04 at the indicated concentrations (red scale bar, 150 μm, yellow scale bar, 300 μm). *n* = 3 biologically independent experiments, one-way ANOVA with Tukey’s multiple comparison test. The values are expressed as the means ± SEMs; *p < 0.05, **p < 0.01, and ***p < 0.001.

We further verified that the mRNA levels of selected genes in the stem cell population maintenance pathway were regulated by ARID3A expression (Figs. 4A and S4A). Importantly, ARID3A enrichment at the promoter regions of these genes was confirmed by ChIP-qPCR (Fig. S4B). We next found that the mRNA level of ARID3A was significantly correlated with those of SMC3 and KDM3A (Spearman correlation coefficient (*r*) > 0.5) in TCGA-LIHC patients (Figs. 4B, C and S4C–F). Importantly, by analysing ChIP-seq data for SMC3 and KDM3A in the liver cancer cell line HepG2, we found that the number of KDM3A binding peaks located at TSS regions of ARID3A-associated ES genes was much greater than the number of SMC3 binding peaks (Figs. 4D and S4G). These results suggested that ARID3A could directly regulate the expression of KDM3A and might further affect the expression of downstream ARID3A-associated ES genes.

KDM3A, a histone demethylase, promotes the transcription of downstream genes through H3K9me1/2 demethylation [18]. We confirmed that overexpression of ARID3A caused a significant increase in the protein level of KDM3A, while ARID3A knockdown reduced KDM3A expression (Figs. 4E and S4H). And KDM3A expression also significantly increased in the xenografts from the ARID3A overexpression group (Fig. S4I). We next silenced KDM3A with siRNAs in Huh7 cells and performed RNA-seq. We found that over half of the KDM3A-bound ARID3A-associated ES genes were downregulated following silencing of KDM3A (Figs. 4F and S4J). Not surprisingly, the promoter regions of most ES signature genes were occupied by KDM3A. Importantly, interference with ARID3A expression reduced KDM3A occupancy at those genes’ promoter regions (Figs. 4G and S4K).

Furthermore, knockdown of KDM3A led to a reduction in the levels of ARID3A-associated ES genes, suggesting an active role for KDM3A in mediating the expression of these genes in liver cancer (Figs. 4H and S4L). Interestingly, the ChIP-seq data demonstrated enrichment of histone H3K9me2 modification after ARID3A knockdown (Fig. 4I). Notably, upregulation of ARID3A expression reduced H3K9me2 in ARID3A-associated ES genes, while knockdown of KDM3A rescued this effect (Figs. 4J and S4M).

To investigate the biological function of KDM3A mediated by ARID3A, we first overexpressed ARID3A and then silenced KDM3A in Huh7 and HepG2 cells. The results showed that KDM3A knockdown strikingly decreased the migration, invasion and tumoursphere formation of ARID3A-overexpressing liver cancer cells (Figs. 4K, L and S5A). In contrast, the cell migration, invasion and tumoursphere formation capabilities were obviously restored by KDM3A overexpression in ARID3A-silenced cells (Fig. S5B, C). Moreover, treatment with either of two KDM3A inhibitors, IOX1 and JIB-04, significantly inhibited cell migration, invasion and tumoursphere formation in ARID3A-overexpressing cells (Figs. 4M, N and S5D). Taken together, these results indicate that KDM3A is a direct downstream effector of ARID3A-mediated regulation of ES signature genes.

### ARID3A interacts with CEP131 in liver cancer cells

**Fig. 5:**
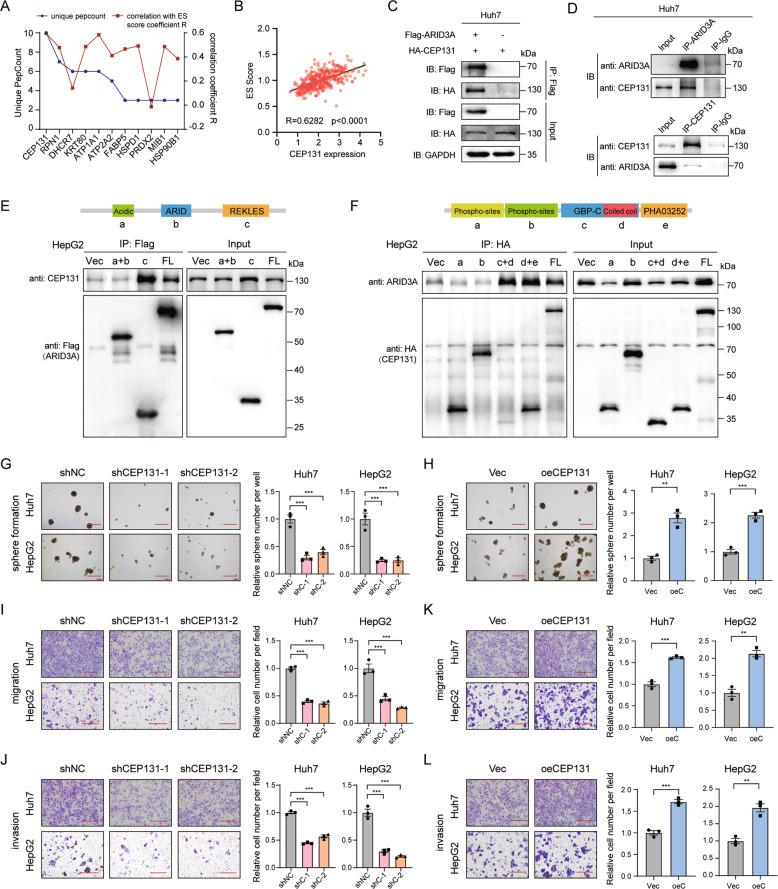
ARID3A interacts with CEP131 in liver cancer cells. **A** Unique peptide counts and ES scores of ARID3A-associated genes identified by Co-IP/LC-MS. **B** Correlations between ES scores of individual patient samples and CEP131 expression levels were determined from TCGA-LIHC data. *n* = 374, Spearman’s rank correlation analysis. **C** Verification of the interaction between exogenously overexpressed ARID3A and CEP131 in Huh7 cells by Co-IP. Anti-Flag antibody-captured CEP131. **D** Verification of the interaction between endogenous ARID3A and CEP131 in Huh7 cells by Co-IP. Top, anti-ARID3A antibody-captured CEP131; bottom, anti-CEP131 antibody-captured ARID3A. **E**, **F** Schematic diagrams of the ARID3A (**E**) and CEP131 (**F**) truncation mutants based on their structural domains. Western blotting was performed to determine the immunoprecipitation efficiency of Flag-tagged ARID3A and HA-tagged CEP131 in HepG2 cells. **G**, **H** Tumoursphere formation assays of liver cancer cells after CEP131 knockdown (shNC as the control) (**G**) and overexpression (Vec as the control) (**H**) (scale bar, 300 μm). *n* = 3 biologically independent experiments, one-way ANOVA with Tukey’s multiple comparison test or two-tailed Student’s *t*-test. **I**–**L** Transwell migration and invasion assays of liver cancer cells with CEP131 knockdown (shNC as the control) (**I**, **J**) and overexpression (Vec as the control) (**K**, **L**) (scale bar, 150 μm). *n* = 3 biologically independent experiments, one-way ANOVA with Tukey’s multiple comparison test or two-tailed Student’s *t*-test.

To identify the cofactor that interacts with ARID3A, we performed a Co-IP assay followed by LC-MS in Huh7 cells. we obtained 60 potential interacting proteins base on the criteria of a number of unique peptides > 1 with no IgG-specific peptides (Table S3). Among them, centrosomal protein 131 kDa (CEP131; also called AZI1) ranked first based on the number of unique peptides counts and the correlation with the ES score (Fig. 5A, B). Subsequently, the interaction between ARID3A and CEP131 was confirmed by Co-IP in Huh7 and HEK-293T cells transfected with Flag-tagged ARID3A and HA-tagged CEP131 (Figs. 5C and S6A). Furthermore, we determined by Co-IP that endogenous ARID3A can interact with CEP131 in liver cancer cells (Fig. 5D).

ARID3A consists of an acidic region, an ARID domain, and a REKLES domain (Fig. 5E). Using a series of Huh7 and HepG2 cells expressed ARID3A/CEP131 deletion mutant proteins, we found that the REKLES domain of ARID3A, which is reported to regulate its nucleoplasmic shuttling, and the coiled–coil region in the GBP domain of CEP131 are necessary for their interaction (Fig. 5E, F and Fig. S6B, C) [19, 20]. Next, we investigated the biological function of CEP131 in liver cancer. Similar to the effects of silencing ARID3A expression, silencing the expression of CEP131 reduced the cell migration, invasion and tumoursphere formation capabilities, while overexpression of CEP131 enhanced these capabilities in liver cancer cells (Fig. 5G–L).

### ARID3A and CEP131 coactivate *KDM3A* transcription to regulate the ES signature genes

**Fig. 6:**
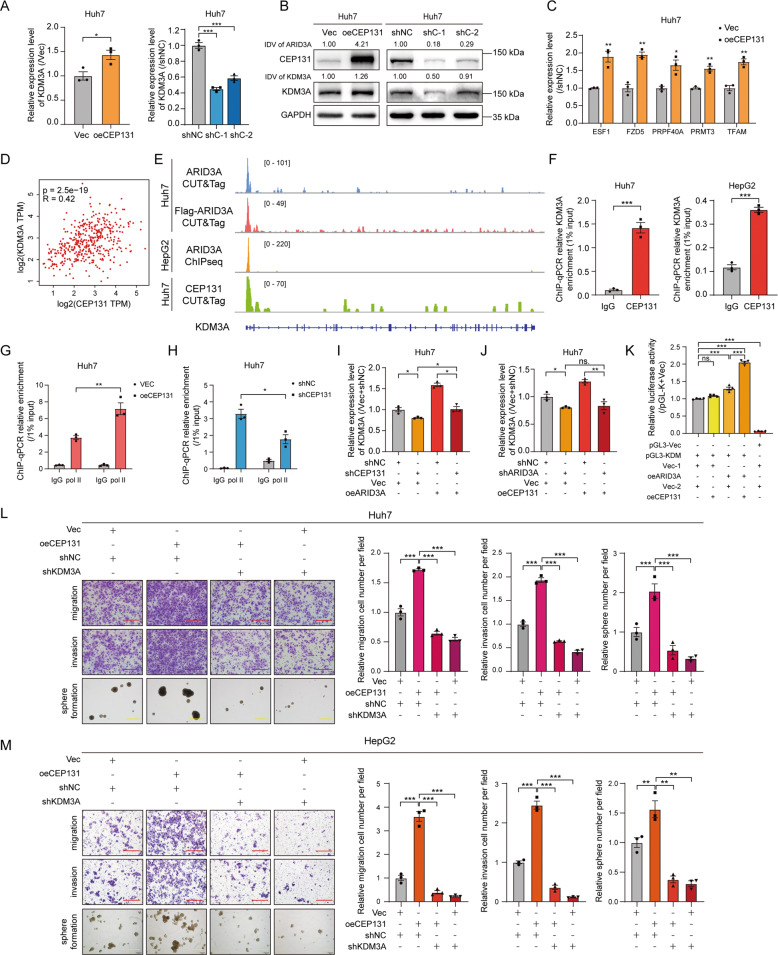
ARID3A and CEP131 cooperatively activate KDM3A transcription to regulate ES signature genes. **A**, **B** The mRNA (**A**) and protein (**B**) expression levels of KDM3A were determined by qPCR and western blot analysis, respectively, in Huh7 cells after CEP131 overexpression or knockdown. *n* = 3 biologically independent experiments, two-tailed Student’s *t*-test or one-way ANOVA with Tukey’s multiple comparison test. The integrated density value (IDV) was calculated by ImageJ and normalized to the internal control. **C** The mRNA levels of representative ARID3A-associated ES genes in CEP131-overexpressing Huh7 cells. *n* = 3 biologically independent experiments, two-tailed Student’s *t*-test. **D** Correlations between the expression of KDM3A and CEP131 in TCGA-LIHC (tumour and normal) samples were determined from the GEPIA database by Spearman’s rank correlation analysis. **E** Profiles of CEP131 and ARID3A occupancy at the promoter region of KDM3A in Huh7 and HepG2 cells. **F** CEP131 occupancy at the KDM3A genomic locus was detected by ChIP-qPCR in Huh7 and HepG2 cells. *n* = 3 biologically independent experiments, two-tailed Student’s *t*-test. **G**, **H** Pol II occupancy levels at the KDM3A promoter were determined by ChIP-qPCR after CEP131 overexpression (**G**) or knockdown (**H**). *n* = 3 biologically independent experiments, two-tailed Student’s *t-*test. **I**, **J** The relative expression level of KDM3A was determined by qPCR after the indicated treatments. *n* = 3 biologically independent experiments, one-way ANOVA with Tukey’s multiple comparison test. **K** Luciferase reporter assay of KDM3A promoter activity in HEK-293T cells after the indicated treatments. *n* = 4 biologically independent experiments, one-way ANOVA with Tukey’s multiple comparison test. **L**, **M** Transwell and tumoursphere formation assays were performed in Huh7 and HepG2 cells treated as indicated. *n* = 3 biologically independent experiments, one-way ANOVA with Tukey’s multiple comparison test (red scale bar, 150 μm, yellow scale bar, 300 μm). The values are expressed as the means ± SEMs; *p < 0.05, **p < 0.01, and ***p < 0.001.

To explore whether KDM3A expression is regulated by CEP131, we overexpressed and knocked down CEP131 in Huh7 cells. Both the mRNA and protein levels of KDM3A were increased upon CEP131 overexpression, while knockdown of CEP131 reduced KDM3A expression, suggesting that KDM3A is also a downstream target of CEP131 in liver cancer cells (Fig. 6A, B). In addition, we found that overexpression of CEP131 significantly increased the mRNA levels of ARID3A-associated ES genes, while knockdown of CEP131 expression reduced the levels of these genes (Figs. 6C and S7A). Furthermore, the mRNA level of KDM3A correlated significantly with that of CEP131 in liver cancer patients based on data from the TCGA-LIHC cohort (Fig. 6D). To explore the mechanism by which KDM3A is regulated by CEP131, we also performed CUT&Tag and found the TSS region of KDM3A was occupied by CEP131; this region was also occupied by ARID3A, as shown by previous results (Fig. 6E). Then CEP131 enrichment was confirmed at the TSS of *KDM3A* in Huh7 and HepG2 cells (Fig. 6F). Importantly, overexpression of CEP131 increased Pol II occupancy at the promoter region of KDM3A, while interference with CEP131 expression reduced Pol II occupancy (Fig. 6G, H). These findings suggest that CEP131 can also regulate the promoter activity of KDM3A and further modulate the expression of ARID3A-associated ES genes.

To further elucidate the underlying mechanisms by which the ARID3A/CEP131 complex induces KDM3A expression, we carried out rescue experiments. Overexpression of either ARID3A or CEP131-induced KDM3A mRNA expression in liver cancer cells, and this induction was abolished by silencing either CEP131 or ARID3A (Fig. 6I, J). Importantly, ARID3A was required for CEP131-induced KDM3A upregulation. The results of luciferase reporter assays confirmed that KDM3A promoter activity was significantly enhanced in the presence of ARID3A (Fig. S7B, C). Intriguingly, simultaneous overexpression of ARID3A and CEP131 led to an even more significant induction in the luciferase activity of the KDM3A promoter, and overexpression of CEP131 alone could not significantly induce the luciferase activity of the KDM3A promoter in HEK-293T cells (Fig. 6K). Furthermore, overexpression of CEP131 enhanced the cell migration, invasion and tumoursphere formation capabilities, whereas these effects were reversed by KDM3A knockdown (Fig. 6L, M). Analysis of the clinical expression data in the TCGA-LIHC cohort showed that both CEP131 and KDM3A were upregulated in tumour tissues compared to NT tissues (Fig. S8A, B). Moreover, a high CEP131 or KDM3A level was associated with poor overall survival in liver cancer patients (Fig. S8C, D). Collectively, these results suggest that ARID3A and CEP131 coregulate ES signature through activation of KDM3A.

### High ARID3A expression in liver cancer may be mediated by DNA demethylation

**Fig. 7:**
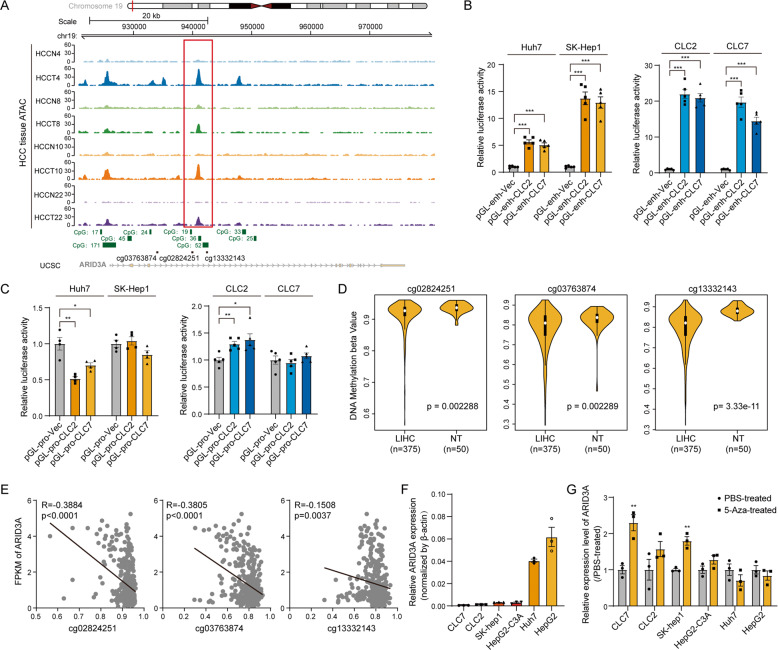
High ARID3A expression in liver cancer may be mediated by DNA demethylation. **A** Profiles of ATAC-seq peaks at the genomic locus of ARID3A in liver cancer tissue from liver cancer patients. The red box indicates the open chromatin region, which is located at the third intron region of ARID3A. **B** Relative luciferase activity in Huh7, SK-Hep1, CLC2, and CLC7 cells after transfection with pGL3-enhancer and pRLTK. *n* = 5 biologically independent experiments, one-way ANOVA with Tukey’s multiple comparison test. **C** Relative luciferase activity in Huh7, SK-Hep1, CLC2, and CLC7 cells after transfection with pGL3-promoter and pRLTK. *n* = 5 biologically independent experiments, one-way ANOVA with Tukey’s multiple comparison test. **D** DNA methylation in the TCGA-LIHC cohort was assessed with the indicated probes in an Illumina Human Methylation 450 array. *n* = 375 liver cancer tissues and 50 adjacent normal tissues, two-tailed Student’s *t-*test. **E** Correlation between DNA methylation and ARID3A expression in TCGA-LIHC data. *n* = 375 liver cancer tissues in the LIHC cohort, Pearson correlation analysis. **F** The expression level of ARID3A in six liver cancer cell lines was determined by qPCR. **G** ARID3A expression after 5-Aza treatment for 5 days was evaluated by qPCR. *n* = 3 biologically independent experiments, two-tailed Student’s *t*-test. The values are expressed as the means ± SEMs; *p < 0.05, **p < 0.01, and ***p < 0.001.

We also investigated the underlying mechanism by which ARID3A is specifically upregulated in liver cancer. The ARID3A oncogene is transcribed from an alternative promoter located in a TE (transposable element) across 15 cancer types, as demonstrated in a previous study by analysis of CAGE-seq data [21]. We performed ATAC-seq in 4 paired liver cancer tissues and matched nontumour tissues to assess open chromatin regions at the ARID3A gene locus and found that a highly accessible chromatin region was located at the third intron region of ARID3A in liver cancer patients (Fig. 7A). We further cloned this region from patient-derived CLC2/7 cell lines and validated the promoter activity of this fragment by luciferase reporter assays (Fig. 7B, C). In addition, the UCSC database revealed several CpG islands located in the open chromatin region in the promoter of ARID3A; therefore, we hypothesized that this specific promoter is probably related to DNA methylation (Fig. 7A). By investigation of the DNA methylation status in the TCGA-LIHC dataset, 13 probes were found covering this open genomic locus in the Illumina Human Methylation 450 array. Three of these sites (cg02824251, cg03763874 and cg13332143) were significantly demethylated in LIHC tissues compared to adjacent nontumour tissues (Fig. 7D). In addition, ARID3A expression was negatively correlated with DNA methylation at these sites (Fig. 7E). Moreover, we used 5-AZA to treat different cells and found that ARID3A can be upregulated by DNA demethylation in cell lines with low background expression (Fig. 7F, G). Taken together, these data suggest that the increased ARID3A expression in liver cancer may be associated with DNA demethylation and open chromatin accessibility at the ARID3A genomic locus.

## Discussion

ARID3A, also called BRIGHT, is a transcription factor in the ARID family. It has been reported to block cell differentiation and promote proliferation and metastasis [22–25]. However, the role of ARID3A in tumorigenesis and stemness regulation remains controversial [9, 26–31]. The complex composed of ARID3B and ARID3A is directly repressed by Let-7 in head and neck cancer, modulates H3K9me3 at stemness genes, and further regulates cancer stemness [23]. RNA sequencing data revealed the ARID3A/B complex-induced genes involved in cancer and stem cell processes in ovarian cancer cells [22]. In contrast, a recent study showed that the expression of ARID3A assures megakaryocytic differentiation and growth restriction, playing a tumour suppressor role in AMKL [27]. In the present study, we found that ARID3A directly binds to the genomic loci of stem cell maintenance genes and regulates their expression, further positively promoting the expression of ARID3A-associated ES signature genes and eventually playing an oncogenic role in liver cancer progression. These results indicate that ARID3A may act as a predictive marker for tumour grade and malignancy in liver cancer patients and might be a useful biomarker for precise therapy in the clinic.

Recent studies have shown that KDM3A is a direct target of hypoxia-inducible transcription factors, acting as an oncogene to enhance tumour migration, invasion and stemness [32–34]. For example, KDM3A upregulates the CSC marker DCLK1 by binding to the DCLK1 promoter in pancreatic adenocarcinoma [35]. Overexpressed KDM3A in pancreatic cancer cell line HPNE formed foci and spheres in culture and formed tumours and metastases in mice [35]. And KDM3A also functioned as oncogene to regulate colorectal cancer cell migration and invasion through modulating Epithelial–Mesenchymal Transition (EMT) and matrix metalloproteinases (MMPs) [36]. In the present study, we demonstrated that ARID3A and CEP131 co-occupy the promoter region of KDM3A and contribute to its transcriptional activity, ultimately enhancing liver cancer cell oncogenic ability. Our findings suggest a new mechanism by which KDM3A functions as a critical and directly regulated downstream target of ARID3A. KDM3A demethylates mainly the repressive mark H3K9me2 in liver cancer cells.

**Fig. 8:**
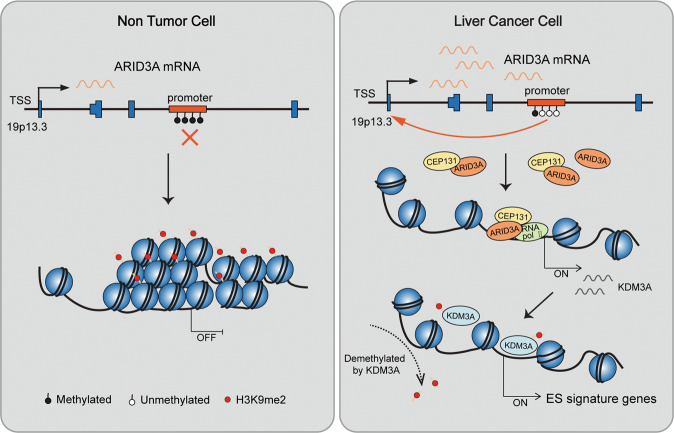
Schematic overview of the mechanism of ARID3A in regulation of ES signature gene in liver cancer. ARID3A has a relative low expression in normal liver cells. We found that ARID3A is upregulated in liver cancer patients, which is at least in part mediated by DNA demethylation at the *ARID3A* gene locus (19p13.3, chromosome 19, p arm, band 1, sub-band 3, and sub-sub-band 3). ARID3A interacts with CEP131 and co-occupies the *KDM3A* promoter region and drive the expression of KDM3A, which demethylates H3K9me2 in the promoter regions of ES signature genes. These events lead to upregulation of ES signature gene expression, facilitating liver cancer malignancy.

In conclusion, our data summarized herein point to a central role of ARID3A in the transcriptional regulation of ES signature genes. ARID3A expression is induced in hepatocarcinoma. ARID3A interacts with CEP131 and co-occupies the *KDM3A* promoter region to activate KDM3A transcription, thereby modulating the expression of ES signature genes in liver cancer cells (Fig. 8). The newly identified ARID3A/CEP131-KDM3A regulatory circuit provides valuable information for the future development of strategies.

## Materials and methods

### Patients and specimens

This study involved two independent cohorts of patients with liver cancer: cohort II, which included 101 liver cancer patients from Zhongshan Hospital; and cohort III, which included 123 liver cancer patients from Fudan University Shanghai Cancer Center. We obtained snap-frozen tumour tissues and adjacent nontumour liver tissues from patients who had first undergone radical resection of liver cancer. Tumour differentiation was graded using the Edmondson grading system. Clinical staging was performed according to the 6th edition of the AJCC/UICC TNM classification system. Time to recurrence and overall survival were calculated from the date of surgery to the date of the first recurrence and death, respectively. Data were censored at the last follow-up for patients without relapse or upon death. Institutional review board approval was obtained and each patient provided written informed consent.

### Tissue microarray and immunohistochemistry (IHC)

Core samples were obtained from representative regions of each tumour based on hematoxylin and eosin staining. First, place the slides in the xylene and alcohol baths (decreasing concentration) for deparaffinization and hydration. Then stain nuclear in hematoxylin (Sangon, Shanghai, China) for 3 min followed by tap water wash. The slides were dipped in ammonia water until the sections become blue, and stained in 1% Eosin (Sangon, Shanghai, China) for 10 min, followed by dehydration and clearing. Then observe tissues under microscope.

Duplicate 1-mm cores were taken from different areas of the same tissue block for each case (intratumoral tissue and peritumoral tissue). Serial sections (4 μm thick) were placed on slides coated with 3-aminopropyltriethoxysilane. IHC experiments were performed to determine the pathological significance of ARID3A. After blocking of endogenous peroxidase activity and antigen retrieval, the sections were incubated with an anti-ARID3A antibody (Santa Cruz, Texas, USA), followed by the DAB reaction. The staining sections were then reviewed and scored by a pathologist as following rules: negative staining (−, scored 1, cells with < 10% staining); weak positive staining (+, scored 2, cells with 10–49% staining); medium positive staining (++, scored 3, cells with 50–74% staining); strong positive staining (+++, scored 4, cells with 75–100% staining). The staining colour intensity was scored as light-yellow particle (1), brown-yellow particle (2) and brown particle (3). The final score was defined as staining number score multiplied by staining intensity score. A score of 0–5 was defined negative, and a score exceeded five was defined positive.

### Xenograft in nude mice

For in vivo metastasis assays, male BALB/c nude mice, aged 5–6 weeks old, were housed in laminar flow cabinets under specific pathogen-free conditions with food and water provided ad libitum. Mice (15 in each group) were transplanted with 0.04 mL of cell suspension containing 2 × 10^6^ cells (pWPXL-VECTOR and pWPXL-ARID3A stable Huh7 cell line) mixed with Matrigel/DMEM (Corning, NY, USA) into the livers. After 2 weeks, the mice were sacrificed, and the livers were immediately fixed in 10% neutral PB-buffered formalin for further haematoxylin and eosin (HE) and IHC staining. The metastatic nodules were counted after HE staining.

To further investigate the effect of ARID3A on tumour viability in vivo, a total of 5 × 10^5^ Huh7 cells were subcutaneously injected into the right flank of the axilla of each male BALB/c mouse (six in each group). The mice were sacrificed 4 weeks later. A small piece of fresh tumour tissue was collected into TRIzol reagent (Invitrogen, Carlsbad, CA, USA) for RNA extraction. Mice were randomly allocated to experimental groups and no blinding method was used for injection. There was no animal exclusion criteria. Animal experiments followed the Guide for the Institutional Animal Care and Use Committee (IACUC) of Fudan University Shanghai Cancer Center.

### RNA-seq analysis

The sequencing library was prepared using an Illumina mRNA-seq sample preparation kit and was then subjected to paired-end sequencing on the Illumina HiSeq platform. The paired-end reads were aligned to hg38 using STAR. Bam files obtained from STAR were first converted into bedGraph format using BEDTools and were then converted into BigWig files for visualization with UCSC Genome Browser or IGV. After calculating raw gene expression levels (raw read counts) with FeatureCounts, normalized profiling data were obtained with DESeq2 for differential expression analysis. DEGs were identified as those with an absolute fold change > 1.5.

### Classification of ES score for each tumour sample

Some of the key regulators of ES cell identity (Oct4, Sox2 and Nanog) are expressed only in specific human cancer types. Therefore, detection of the activity of stem-cell regulatory networks must rely on expression analysis of many genes in multiple cancer samples. Previous study analyzed the enrichment patterns of gene sets associated with ES cell identity in the expression profiles of various human tumour types, and found that ES gene sets can reflect ES cell identity [12]. The ES score for each tumour sample was calculated by using gene set variation analysis based on 380 ES signature genes’ expression that contribute to stem cell-like phenotypes shown by many tumours [37]. Spearman’s rank correlation was used to assess the correlation among ES scores based on different expression of genes.

### ChIP and CUT&Tag

Liver cancer cells were crosslinked for 10 min with 1% formaldehyde, followed by the addition of 0.125 M glycine to terminate crosslinking. Then washed and gathered the cells into new tubes. Chromatin was digested into DNA fragments using 0.5 μL Micrococcal Nuclease. The complex of protein and DNA was extracted and resuspended in SimpleChIP Chromatin IP buffers (Cell Signaling Technology, Danvers, Massachusetts, USA). Immunoprecipitation steps were similar with Co-IP. The target DNA were washed and eluted using MinElute Spin Columns (Qiagen, Hilden, Germany) for qPCR examination or DNA sequencing. The primers used in the ChIP-qPCR assay are listed in Table S4. CUT&Tag was performed according to the manufacturer’s protocol (Novoprotein, Shanghai, China). A number of 1 × 10^5^ (Huh7) cells were collected and Incubated with NovoNGS® ConA Beads. Then antibodies and ChiTag transposons were bond to the chromatin in order. After that, DNA fragments obtained, amplified and purified according to the NovoNGS® CUT&Tag High‐Sensitivity Kit.

For ChIP-seq and CUT&Tag analyses, 150-bp paired-end reads were aligned to the reference human genome using Bowtie with standard alignment parameters. PCR duplicates were marked with the Picard “Mark Duplicates” utility and removed from further analysis. Bam files were converted to BigWig files using deepTools. Peaks were identified using the MACS2 peak caller, and then were annotated with Homer. The peak distribution along genomic regions of genes of interest were visualized with IGV.

### Statistical analysis

All data were presented as the mean ± standard error of the mean (SEM). Differences between two groups were assessed with the two-tailed Student’s *t*-test. The one-way analysis of variance (ANOVA) with Tukey’s multiple comparison test was used to assess differences between more than two groups. Each experiment was performed with at least three times. The *χ*2-test was performed to analyse the relationships between ARID3A expression and clinicopathologic factors of the patients. Correlations between KDM3A and ARID3A or CEP131 were analyzed by Spearman’s rank correlation. Overall survival of ARID3A was calculated using the Kaplan–Meier method (The high/low expression of ARID3A was calculated by the ROC curve). No statistical methods were used to predetermine the sample size. No blinding method was used during the experiment. Mice were randomly allocated to experimental groups. There was no animal exclusion criteria. The variance was similar between the groups that were being statistically compared. Statistical analyses and graphical depiction were performed by GraphPad Prism 8 (GraphPad Software, CA, USA), SPSS (IBM, NY, USA) and R project (https://www.r-project.org/). A *p*-value < 0.05 was considered statistically significant.

## Supplementary information

Supporting Information

Academic Journals Reporting Checklist

Full length upcropped original western blots

## Data Availability

ARID3A ChIP-seq (ENCSR000EDP), KDM3A ChIP-seq (ENCODE: ENCSR387JKT) and SMC3 ChIP-seq (ENCODE: ENCSR000EDW) of hepG2 cells were obtained from ENCODE (https://www.encodeproject.org/). Illumina Human Methylation 450 platform-based beta value of DNA methylation in TCGA-LIHC was downloaded from Xena (https://xenabrowser.net/). Other data supporting the finding of the current study are listed in NCBI Gene Expression Omnibus (Accession no: GSE184797 and GSE193726).
