# Supplementary material for: Hepatic ARID3A facilitates liver cancer malignancy by cooperating with CEP131 to regulate an embryonic stem cell-like gene signature
Source: Cell Death Dis. 2022 Aug 25;13(8):732. doi: 10.1038/s41419-022-05187-9 (PMC9411159; doi:10.1038/s41419-022-05187-9)
Supplement: Supplementary file 1 — Supporting Information [file 41419_2022_5187_MOESM1_ESM.docx]

**Supplementary Information**

**Hepatic ARID3A facilitates liver cancer malignancy by cooperating with CEP131 to regulate an embryonic stem cell-like gene signature**

Mengting Shen^1*^, Shengli Li^2*^, Yiming Zhao^3*^, Yizhe Liu^1^, Zhen Liu^1^, Lin Huan^1^, Yejun Qiao^1^, Lu Wang^3#^, Leng Han^4#^, Zhiao Chen^1,5,6#^ and Xianghuo He^1,5,6#^

^1^Fudan University Shanghai Cancer Center and Institutes of Biomedical Sciences; Shanghai Medical College, Fudan University, Shanghai 200032, China.

^2^Precision Research Center for Refractory Diseases, Institute for Clinical Research, Shanghai General Hospital, Shanghai Jiao Tong University School of Medicine, Shanghai 201620, China.

^3^Department of Hepatic Surgery, Fudan University Shanghai Cancer Center, Fudan University, Shanghai 200032, China.

^4^Center for Epigenetics and Disease Prevention, Institute of Biosciences and Technology, Texas A&M University, Houston, TX 77030, USA.

^5^Key Laboratory of Breast Cancer in Shanghai, Fudan University Shanghai Cancer Center, Fudan University, Shanghai 200032, China.

^6^Shanghai Key Laboratory of Radiation Oncology, Fudan University Shanghai Cancer Center, Fudan University, Shanghai 200032, China.

^*^These authors contributed equally to this work.

^#^ **Corresponding Authors:**

Xianghuo He, Email: xhhe@fudan.edu.cn or Zhiao Chen, Email: zachen@fudan.edu.cn, Fudan University Shanghai Cancer Center and Institutes of Biomedical Sciences; Shanghai Medical College, Fudan University, 302 Rm., 7# Bldg., 270 Dong An Road, Shanghai 200032, China. Tel: 86-21-34777329; Fax: 86-21-64172585. Or Leng Han, Email: leng.han@tamu.edu, Center for Epigenetics and Disease Prevention, Institute of Biosciences and Technology, Texas A&M University. 2121 W. Holcombe Blvd, Houston, TX 77030, USA. Or Lu Wang, wangluzl@fudan.edu.cn; Department of Hepatic Surgery, Fudan University Shanghai Cancer Center, Fudan University, 4F., 5# Bldg., 270 Dong An Road, Shanghai 200032, China. Tel: 86-21-64175590.

**Key words**

ARID3A, KDM3A, CEP131, Transcriptional dysregulation, Hepatocarcinoma

**Conflict of Interest**

The authors declare no conflict of interest.

**Supplementary** **Materials and Methods**

**Characterize the expression alteration in TCGA-LIHC**

The mRNA expression profiles and clinical features of HCC patients were downloaded from TCGA data portal (http://gdac.broadinstitute.org/). Normalized gene expression data based on expectation maximization (RSEM). We used Student’s t test to assess the differential expression between TCGA tumour and paired normal samples and followed by FDR adjustment for *p* value. Genes were considered differential expression between tumour and normal paired samples if the fold-change > 1.5 and *t* test FDR < 0.05.

**Cell culture**

HepG2 (ATCC, ATCC Number: HB-8065), SK-hep-1 (ATCC, ATCC Number: HTB-52) and HepG2-C3A (ATCC, ATCC Number: CRL-10741) and HEK293T (ATCC, ATCC Number: CRL-11268) cell lines were obtained from the American Type Culture Collection (Manassas, Virginia, USA). Huh7 cell line was purchased from the Shanghai Cell Bank Type Culture Collection Committee (CBTCCC, Shanghai, China). Cells above were cultured in DMEM medium supplemented with 10% fetal bovine serum (FBS), 100 U/ml penicillin and 100 μg/ml streptomycin (Invitrogen). Patient-derived liver cancer cells CLC2 and CLC7 cells were gifts from laboratory of Hui (Chinese Academy of Sciences)**^1^**, and were cultured in primary culture medium-RPMI1640 medium supplemented with 10% FBS, 110 μg/mL sodium pyruvate, 10 μg/mL insulin, 5.5 μg/mL transferrin, 6.7 ng/mL sodium selenite and 40 ng/mL EGF (Gibco). All cells were cultured at 37°C with a 5% CO_2_ atmosphere. Cells were assessed for mycoplasma monthly via the qPCR analysis using specific primers for detecting mycoplasma (Table S4). All these cells were recently authenticated by STR analysis.

**Vector construction and lentivirus production**

The entire sequence of ARID3A, CEP131 and KDM3A were amplified from cell cDNA and cloned into the pCDH-Puro vector or pWPXL vector, which was obtained from Addgene (http://www. addgene.org). shRNA sequences were designed in BLOCK-iT™ RNAi Designer (http://rnaidesigner.thermofisher.com/) and inserted into the LentiGuide-Puro lentiviral vector. Relative empty vectors were used as the control. For cell functional screening on TFs, small interfering RNA (siRNA) oligonucleotides were synthesized from RiboBio (RiboBio, Biotechnology, Guangzhou, China). Relative siNC as a negative control. The sequences of siRNAs and primers used in this study are listed in Table S5 and S6.

Cells were seeded in each well of a 6/12/24-well plate at the cell density of 50%-70%. A transfection assay was done using Lipofectamine 2000 or RNAiMAX (Invitrogen) with corresponding vectors or siRNA according to the manufacturer's protocol. For lentiviruses generation, target vector, packaging plasmid (pAX2), and VSV-G envelope plasmid (pMD2.G) were cotransfected into HEK-293T cells. And lentiviruses were collected after 48 hours to infect liver cancer cell lines.

**Cell viability assay, migration and invasion assay**

Viability of liver cancer cells were detected in 2D (CCK-8 assay) and 3D culture (sphere formation assay), respectively. Cells were seeded in 96-well pates at the density of 1,000 cells per well. After 1 day, 3 days and 5 days, culturing medium was removed, and the mix of new medium and Cell Counting Kit-8 solution (CCK-8, Dojindo, Kumamoto, Japan) was added into wells and incubated for 2 hours. After incubation, the absorbance was measured at 450 nm. For sphere formation assay, 1×10^4^ (Huh7) or 2×10^4^ (HepG2) cells were suspended in defined sphere culture medium (DMEM/F12 supplemented with N2 Plus Supplement, 20 ng/ml FGF, 20 ng/ml EGF, and 1×B27 supplement) on ultra-low-attachment 6-well plates. The number of spheres larger than 50 μm in diameter was counted after 10 days.

Migration and invasion assays were performed using 8-μm pore size chambers (Corning, NY, USA) in 24-well plates. Cells at the density of 5×10^4^ (for migration) or 8×10^4^ (for invasion) were suspended in 200 μL serum-free medium then seeded into the upper chambers. The lower chambers were filled with 500 μL complete medium as a chemoattractant. The invasion assay was conducted similarly with the chambers precoated with Matrigel (BD Biosciences, Franklin Lakes, NJ, USA). After a certain incubation time, cells moved to the basal side of the chamber were fixed with methanol and stained with crystal violet. Then cells of each chamber were imaged and counted in three random fields.

Cell wound healing assay also used to detect cell migration ability influenced by ARID3A expression. Cells were cultured in 6-well plates up to 100% confluent. A scratch was created by scratching cell monolayers with a sterile 200 μl plastic pipette tip. Images of same sites were captured at certain time interval by phase contrast microscopy.

**Fluorescence-activated Cell Sorting (FACS) assay**

Single cells were isolated and collected as a number of 5×10^5^ (Huh7) cells for each sample. For stemness markers staining, single cells were stained with APC-conjugated anti-human CD326 (EpCAM) Antibody or PE-conjugated anti-human CD13 Antibody (dilution at 1:40) at room temperature for 30 min. Flow cytometry analysis and cell sorting were conducted by MOFLO ASTRIOS (Beckman Coulter) instrument and analyzed by Summit 6.3 software. Information on the antibodies was listed in Table S7.

**RNA extraction and quantitative real-time PCR (qPCR)**

The RNA samples of clinical tissue specimens, animal tumour tissues and cell lines were extracted using TRIzol reagent (Invitrogen, CA, USA). Then they were reverse transcribed using the PrimeScript RT Reagent kit (TaKaRa, Tokyo, Japan). SYBR Green Premix Ex Taq (TaKaRa, Tokyo, Japan) was used to perform quantitative Real-Time PCR. RNA expression levels were measured on a 7900 Real- Time PCR System with the SDS 2.3 software sequence detection system (Applied Biosystems, USA). The primers used for qRT-PCR were listed in Table S4. β-actin was used as an internal control.

**Gene Set Enrichment Analysis (GSEA)**

GSEA was conducted by the preranked method using the clusterProfiler package in R (<https://www.r-project.org/>). The RNA-seq gene list and ranks of differences were used for GSEA. The “stem cell population maintenance” gene set (downloaded from the GO website) and “ES cell-like gene expression signature”**^2^** gene set were analyzed to visualize significant enrichment.

**Western blotting analysis**

Cell lysis was boiled for 15 minutes with Omni-Easy™ Protein Sample Loading Buffer (Epizyme, Shanghai, China). The protein concentrations were separated by SDS-PAGE and then transferred to 0.2μm nitrocellulose membranes (GE Healthcare, CT, USA). The membranes were blocked with 5% BSA (Sigma-Aldrich, CA, USA) or 5% non-fat milk for one hour at room temperature. After probing with primary antibodies at 4 °C overnight, the membranes were incubated by HRP-conjugated secondary antibodies. The detection of immune complexes was performed using a LumiBest ECL Reagent Solution kit (Share-Bio, Shanghai, China). Information on the antibodies are listed in Table S7.

**Co-immunoprecipitation (Co-IP)**

The interaction of ARID3A and CEP131 in cell lines was evaluated by Co-IP assay. Cells were collected and lysed in IP lysis buffer, containing 25 mM Tris-HCl (pH 7.4), 150 mM NaCl, 1 mM EDTA, 1% NP-40 and 5% glycerol, supplemented with proteinase inhibitors (Bimake, Shanghai, China). After centrifugation, the supernatants were incubated with the Protein A/G magnetic beads (Bimake, Shanghai, China) combined antibodies: ARID3A, CEP131, FLAG or HA, overnight at 4°C. After incubation, the beads were washed five times with NT2 buffer and were boiled in SDS loading buffer. To find the specific binding domain, truncated ARID3A or CEP131 were amplified from full-length CDS and subcloned into pCDH-3×Flag vector. Then stable cell lines were constructed to perform Co-IP assays. The interaction proteins were separated by SDS-PAGE and detected by Mass Spectrometry (Omicsolution, Shanghai, China) or immunoblotting. Related antibodies and primers are listed in Table S6 and S7.

**Luciferase reporter assays**

Fragments of ARID3A and KDM3A promoter region were amplified from cell genomic DNA and then cloned into pGL3.0-enhancer or pGL3.0-promoter vectors at the KpnI and XhoI sites. The primers were shown in Supplementary Table 6. HEK-293T or Huh7 cells were seeded into 96-well plates and co-transfected with luciferase reporter plasmid pGL3.0 and pRL-TK for 48 hours. Firefly and Renilla luciferase activities were measured using the dual-luciferase reporter assay system (Promega, WI, USA).

**ATAC-seq**

Fresh liver cancer tissues were cut into 1-2 mm pieces and digested using collagenase. Grinding and counting to isolate 50000 living cells. Transpose, purify and PCR steps were performed as previously described^3^. All deep sequencing was performed on the Illumina HiSeq Xten-PE150 or Illumina HiSeq 2500 platform provided by GENEWIZ (GENEWIZ Suzhou, China). The following tools and versions were used for ATAC-seq data analysis: Trimmomatic, SAMtools, Picard, and Bowtie2. First, Nextera adapter sequences were trimmed from the reads by using Trimmomatic. These reads were aligned to a reference genome using Bowtie2 with standard parameters. Picard was then used to remove duplicate reads. These deduplicated reads were then filtered to retain high-quality (MAPQ ≥ 30), non-mitochondrial chromosome, non-Y chromosome, and properly paired (SAMtools flag 0 × 2) reads.

**Reference**

1. Qiu Z, Zou K, Zhuang L, Qin J, Li H, Li C*, et al.* Hepatocellular carcinoma cell lines retain the genomic and transcriptomic landscapes of primary human cancers. *Sci Rep* 2016, **6:** 27411.

2. Ben-Porath I, Thomson MW, Carey VJ, Ge R, Bell GW, Regev A*, et al.* An embryonic stem cell-like gene expression signature in poorly differentiated aggressive human tumors. *Nat Genet* 2008, **40**(5)**:** 499-507.

3. Buenrostro JD, Giresi PG, Zaba LC, Chang HY, Greenleaf WJ. Transposition of native chromatin for fast and sensitive epigenomic profiling of open chromatin, DNA-binding proteins and nucleosome position. *Nat Methods* 2013, **10**(12)**:** 1213-1218.06

**Supplementary Figures**

**Figure S1**


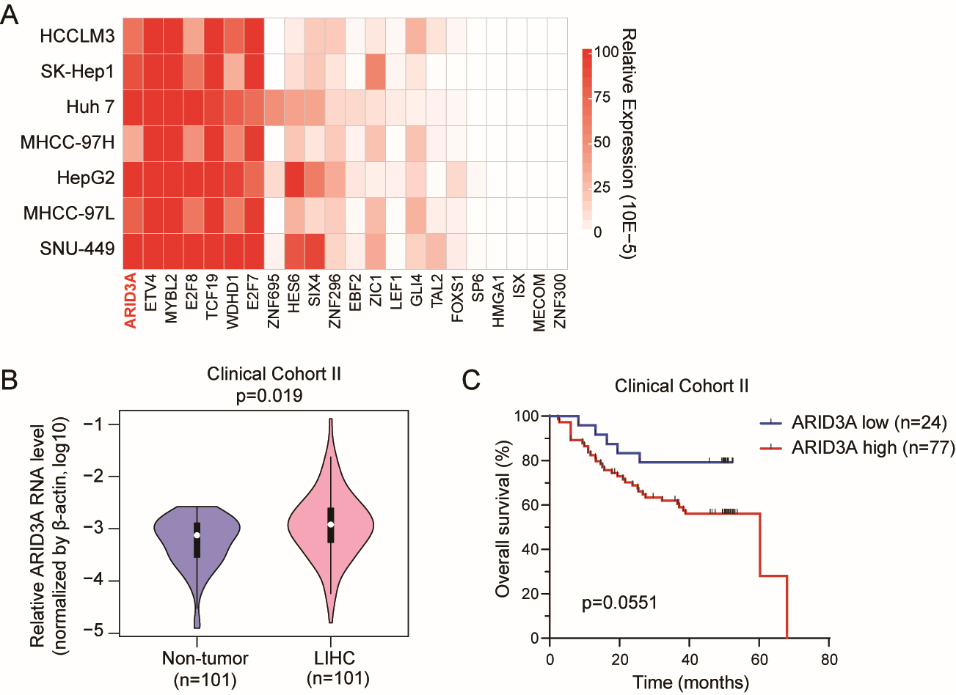


**Fig. S1 High expression of ARID3A correlates with poor outcomes and cancer stemness in liver cancer patients.** **A** Expression profile of 22 selected TF genes in 7 liver cancer cell lines was determined by qPCR. **B** ARID3A expression was increased in tumour tissues in 101 paired samples in the Cohort II, determined by qPCR, two-tailed Student’s *t* test. **C** Kaplan–Meier analysis of overall survival for liver cancer patients in the TCGA-LIHC Cohort II (n = 101), Log-rank test.

**Figure S2**


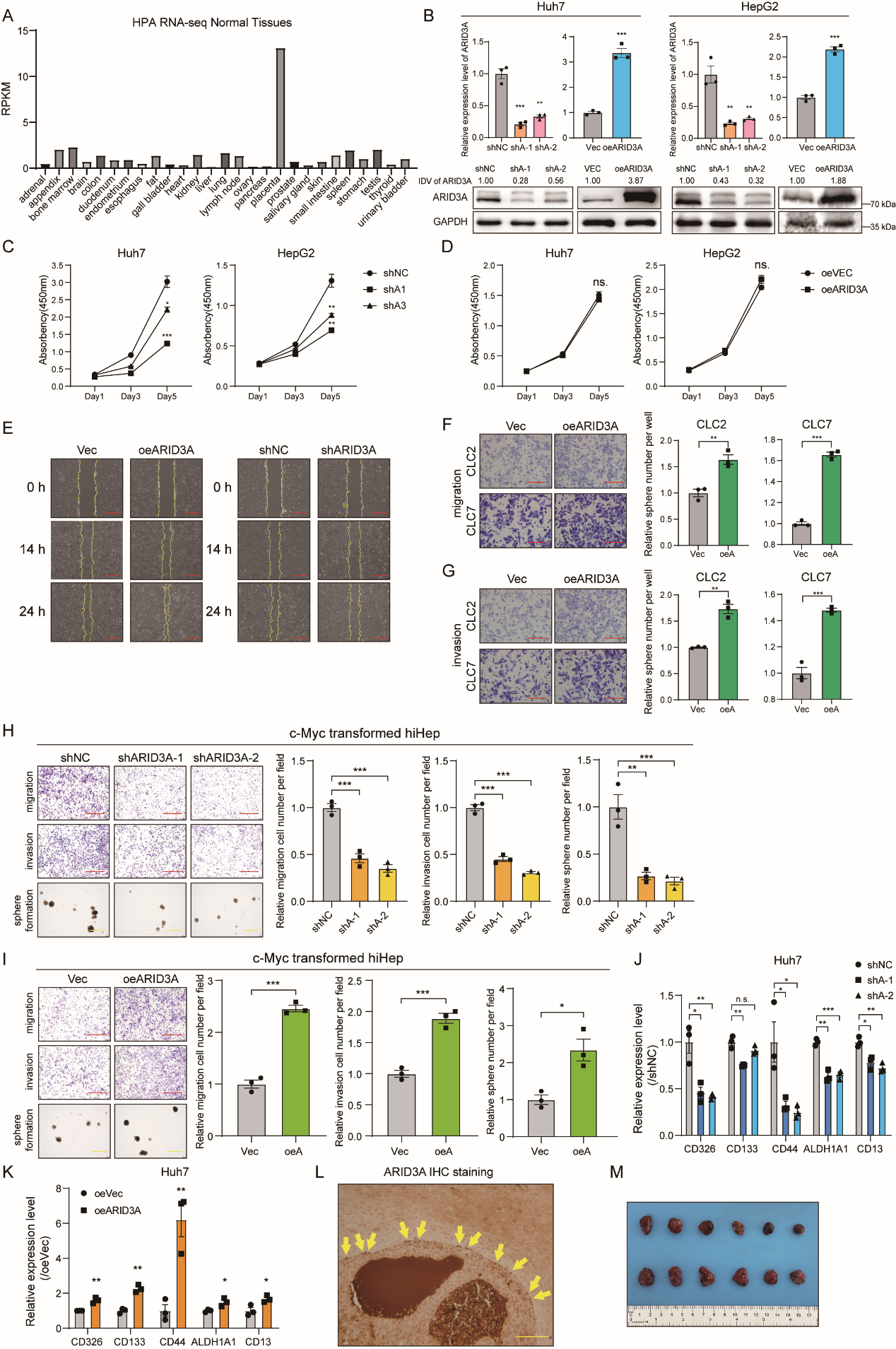


**Fig. S2** **ARID3A promotes liver cancer cell tumoursphere formation, migration, invasion and metastasis.** **A** ARID3A expression level (mean) in normal tissues, generated from HPA. **B** Knockdown and overexpression efficiency of ARID3A in Huh7 and HepG2 cells were determined by qPCR and WB. n=3 biologically independent experiments, one-way ANOVA with Tukey’s multiple comparison test and two-tailed Student’s *t* test. **C and D** CCK8 assays of Huh7 and HepG2 cells after ARID3A knockdown (shNC as the control) **(C)** and ARID3A overexpression (Vec as the control) **(D)**. n = 3 biologically independent experiments, one-way ANOVA with Tukey’s multiple comparison test and two-tailed Student’s *t* test. **E** Wound healing assays of Huh7 cells treated as indicated (scale bar, 300 μm). **F and G** Transwell migration **(F)** and invasion **(G)** assays of CLC2 and CLC7 cells after overexpressing ARID3A (Vec as the control, scale bar, 150 μm). n = 3 biologically independent experiments, two-tailed Student’s *t* test. **H and I** Transwell and tumoursphere formation assays were performed in c-Myc-transformed HiHep cells after ARID3A knockdown **(H)** or overexpression **(I)**. n=3 biologically independent experiments, one-way ANOVA with Tukey’s multiple comparison test and two-tailed Student’s *t* test (red scale bar, 150 μm, yellow scale bar, 300 μm). **J and K** The mRNA levels of liver cancer stemness markers in Huh7 cells after ARID3A knockdown **(J)** or overexpression **(K)**. n=3 biologically independent experiments, one-way ANOVA with Tukey’s multiple comparison test and two-tailed Student’s *t* test. **L** A representative image of ARID3A immunohistochemistry of tumour periphery in mouse liver. Yellow arrows indicate the high expression of ARID3A (scale bar, 300 μm). **M** Representative image of Tumours of xenograft mouse model, control (up) and ARID3A overexpressed (down), n = 6. The values are expressed as the means ± SEMs; *p < 0.05, **p < 0.01, and ***p < 0.001.

**Figure S3**


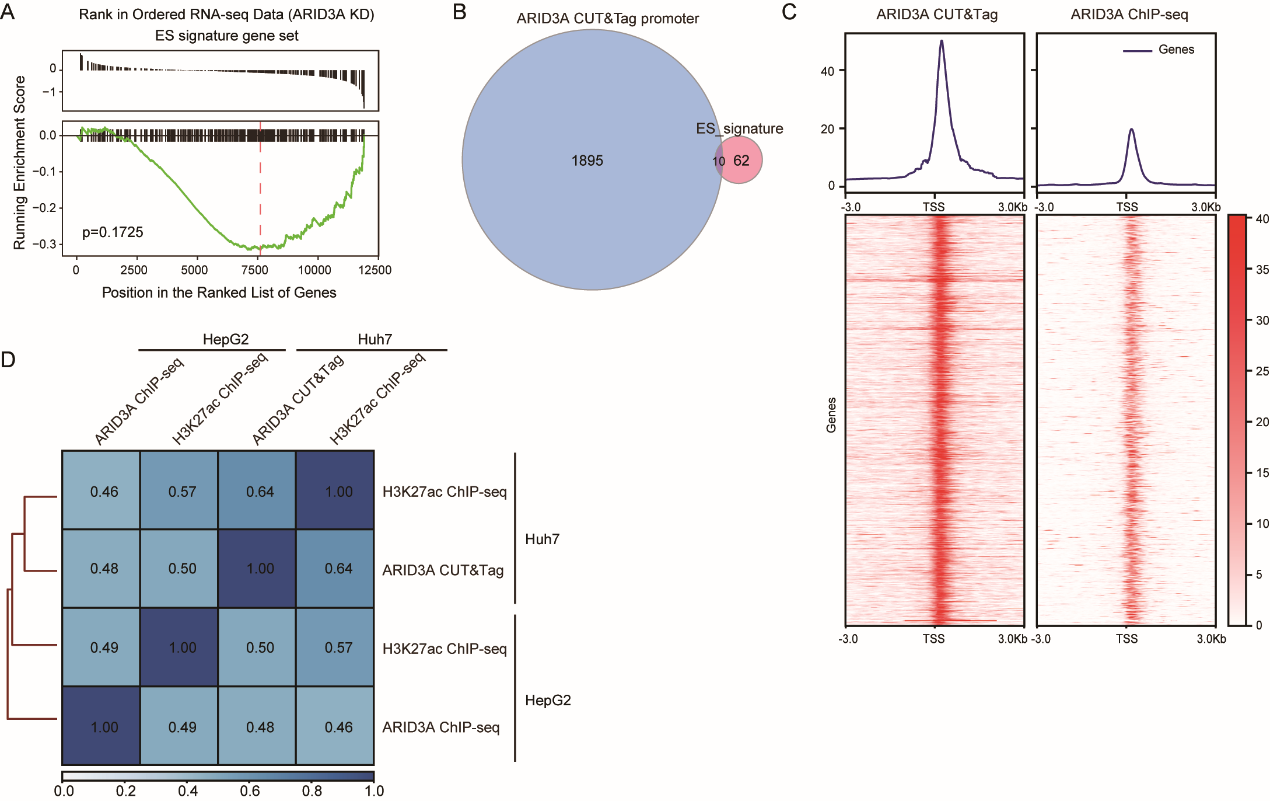


**Fig. S3** **ARID3A regulates the expression of stem cell population maintenance genes.** **A** GSEA of the ES signature gene set in RNA-seq data from Huh7 cells knockdown ARID3A (vs. control vector cells). **B** Overlap of genes corresponding to ARID3A bound promoter sites with ES signature genes. **C** ARID3A genomic occupancy profile from CUT&Tag in Huh7 and ChIP-seq in HepG2. Each row represents a specific genomic locus. **D** Correlations between ARID3A-binding regions identified by ChIP-seq or CUT&Tag and H3K27ac signals.

**Figure S4**


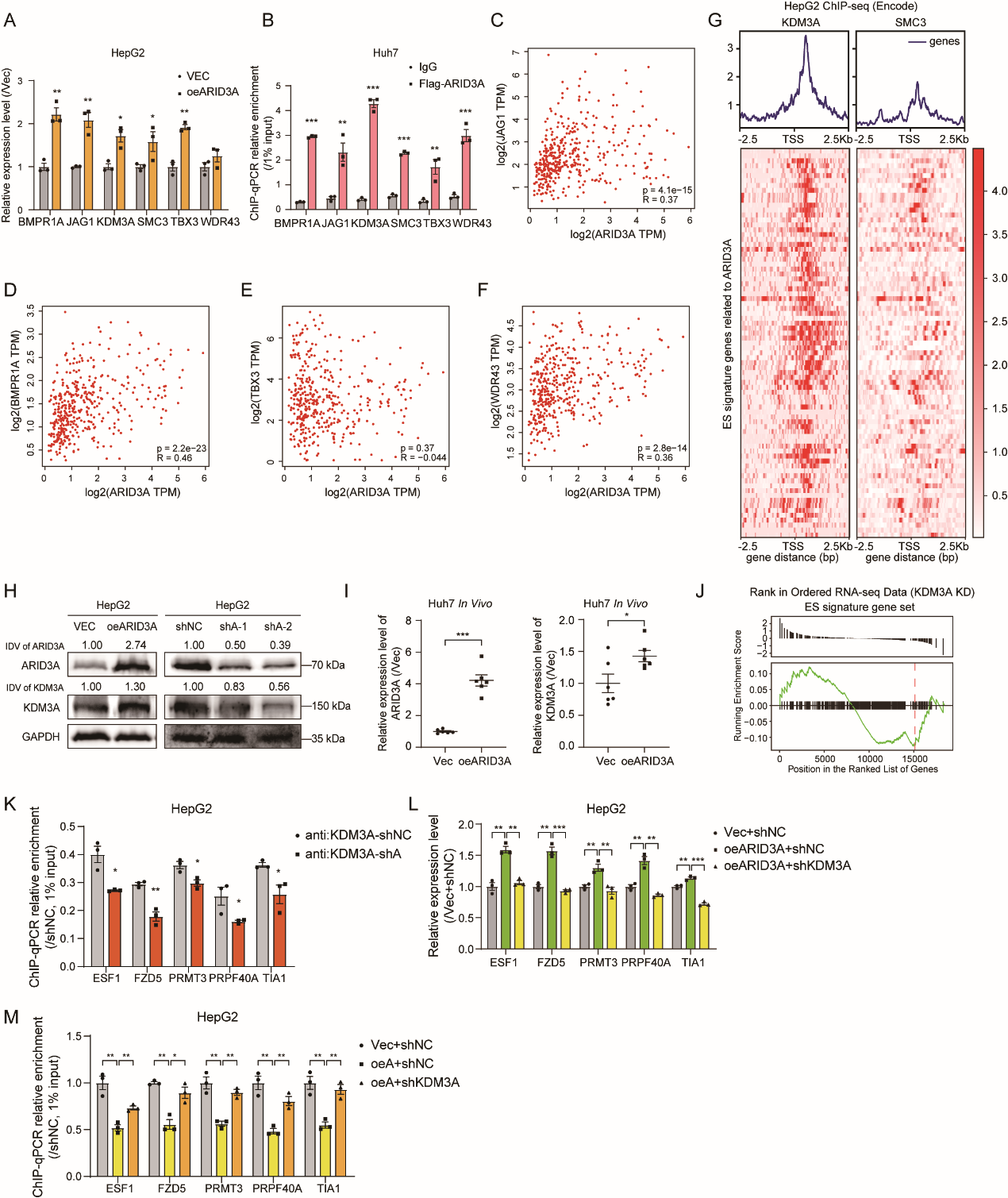


**Fig. S4 ARID3A activates ES signature gene expression through H3K9me2 demethylation by KDM3A**. **A** The mRNA expression levels of stem cell population maintenance genes were determined by qPCR in ARID3A overexpressed HepG2 cells. n=3 biologically independent experiments, two-tailed Student’s *t* test. **B** ARID3A occupancy in genomic loci of stem cell population maintenance genes was detected by ChIP-qPCR. n=3 biologically independent experiments, two-tailed Student’s *t* test. **C-F** Correlations between the expression of stem cell population maintenance genes and ARID3A in TCGA-LIHC (tumour and normal), were determined from the GEPIA database by Spearman’s rank correlation analysis. **G** Genomic occupancy profile of KDM3A (ENCODE: ENCSR387JKT) and SMC3 (ENCODE: ENCSR000EDW) in the TSS regions of the ARID3A-associated ES genes in HepG2 cell ChIP-seq data. Each row represents a specific genomic locus. **H** KDM3A protein expression levels were determined by western blot analysis in HepG2 cells after ARID3A overexpression or knockdown. The integrated density value (IDV) was calculated by ImageJ and normalized to the internal control. **I** The mRNA expression levels of ARID3A and KDM3A in tumours xenograft mouse model. n=6 biologically independent experiments, two-tailed Student’s *t* test. **J** GSEA enrichment for the ES signature gene set in Huh7 cells after KDM3A knockdown (vs. shNC cells). **K** The KDM3A occupancy levels in the promoters of representative ARID3A-associated ES genes were determined by ChIP-qPCR in ARID3A knockdown HepG2 cells (vs. shNC cells). n=3 biologically independent experiments, two-tailed Student’s *t* test. **L** The mRNA levels of representative ARID3A-associated ES genes in HepG2 cells treated as indicated. n=3 biologically independent experiments, one-way ANOVA with Tukey’s multiple comparison test. **M** The H3K9me2 occupancy levels in the promoters of representative ARID3A-associated ES genes were determined by ChIP-qPCR after the indicated treatments. n=3 biologically independent experiments, one-way ANOVA with Tukey’s multiple comparison test. The values are expressed as the means ± SEMs; *p < 0.05, **p < 0.01, and ***p < 0.001.

**Figure S5**


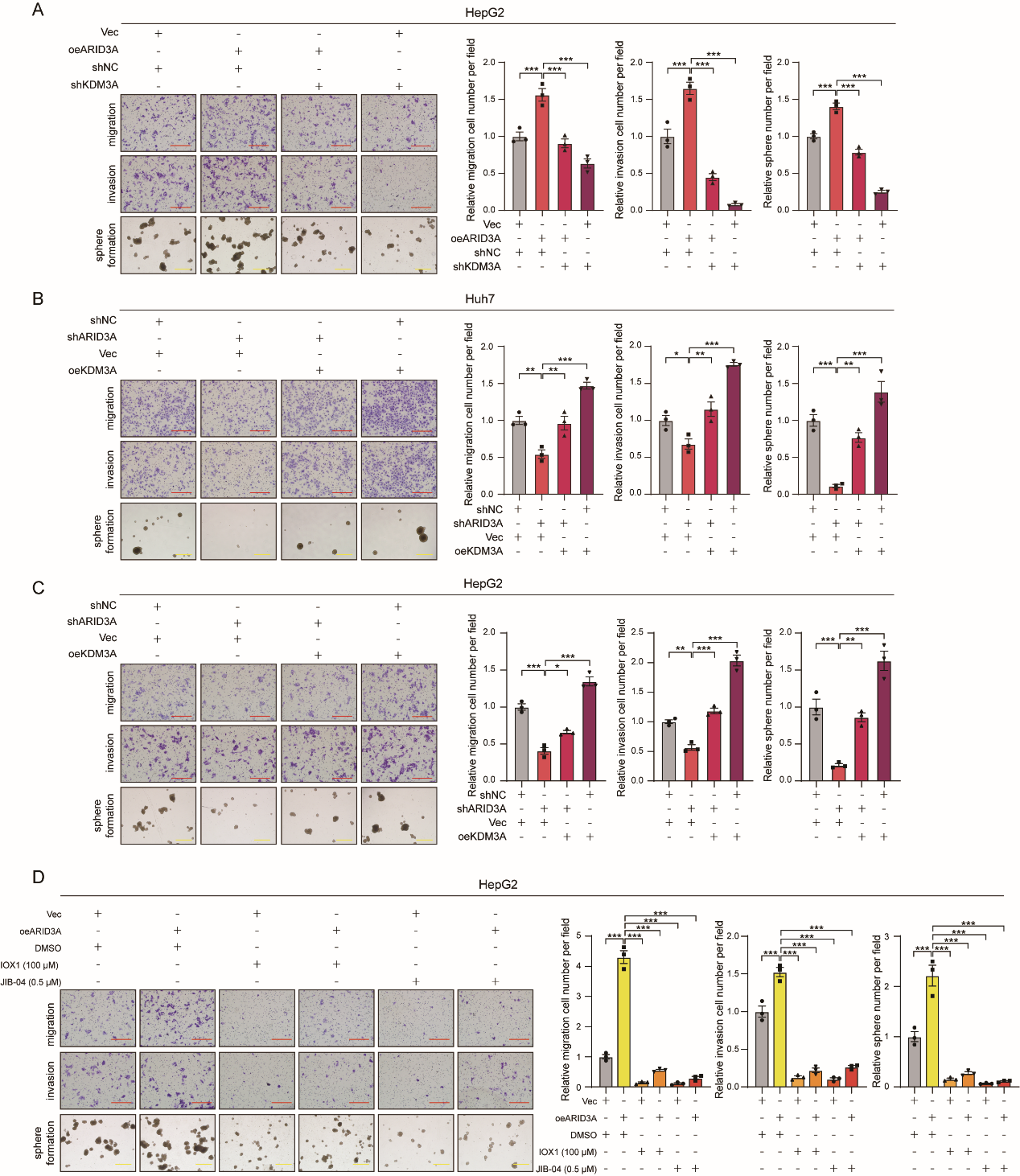


**Fig. S5** **ARID3A-induced tumoursphere formation, cell migration and invasion are mediated by KDM3A.** **A-C** Transwell and tumoursphere formation were performed in Huh7 and HepG2 cells treated as indicated. n=3 biologically independent experiments, one-way ANOVA with Tukey’s multiple comparison test (red scale bar, 150 μm, yellow scale bar, 300 μm). **D** Transwell and tumoursphere formation were performed in ARID3A overexpressed HepG2 cells with KDM3A inhibitors IOX1 and JIB-04 at the indicated concentration (red scale bar, 150 μm, yellow scale bar, 300 μm). n=3 biologically independent experiments, one-way ANOVA with Tukey’s multiple comparison test. The values are expressed as the means ± SEMs; *p < 0.05, **p < 0.01, and ***p < 0.001.

**Figure S6**


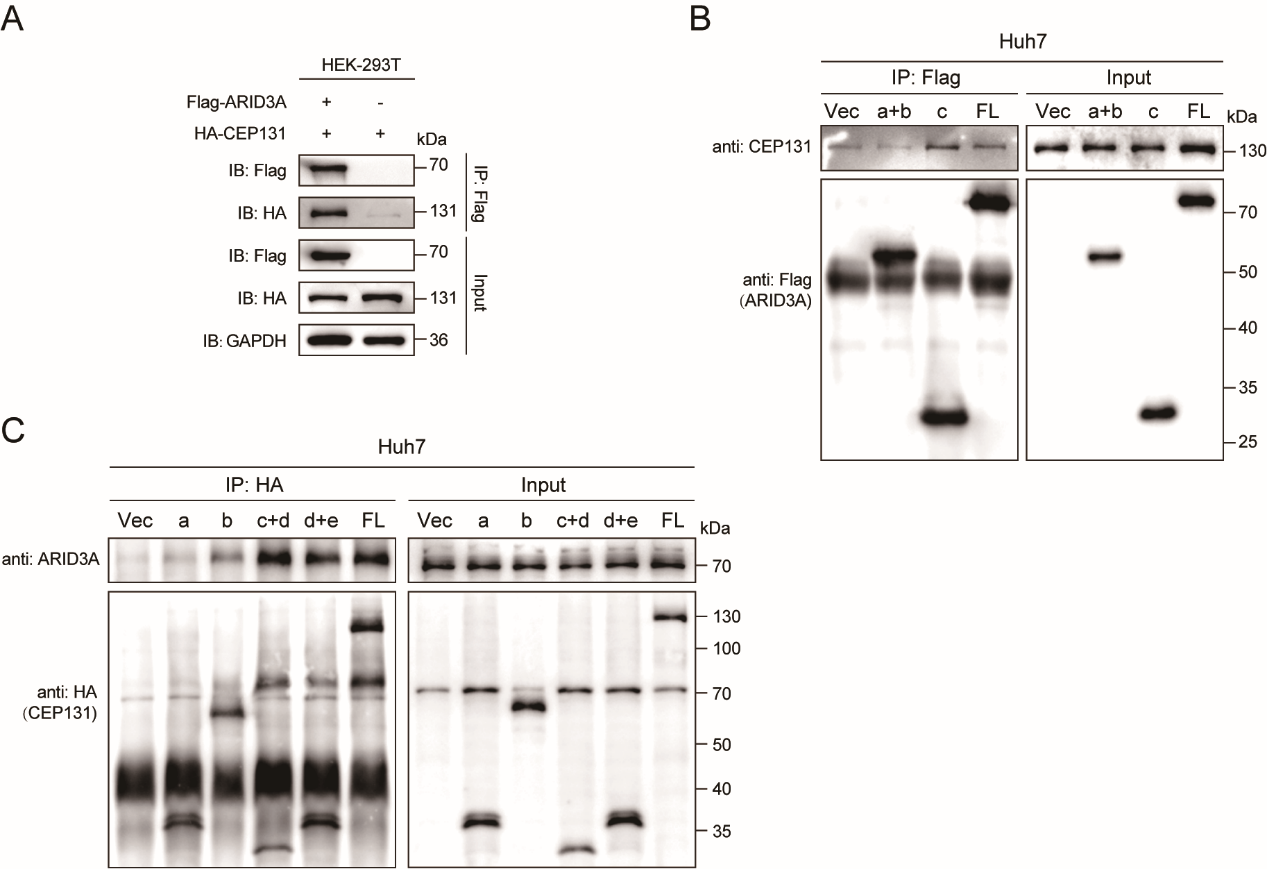


**Fig. S6** **ARID3A interacts with CEP131 in liver cancer cells.** **A** Verification of the interaction between exogenously overexpressed ARID3A and CEP131 in HEK-293T cells by Co-IP. Anti-Flag antibody-captured CEP131. **B and C** Schematic diagrams of the ARID3A **(B)** and CEP131 **(C)** truncation mutants based on their structural domains. Western blotting was performed to determine the immunoprecipitation efficiency of Flag-tagged ARID3A and HA-tagged CEP131 in Huh7 cells.

**Figure S7**


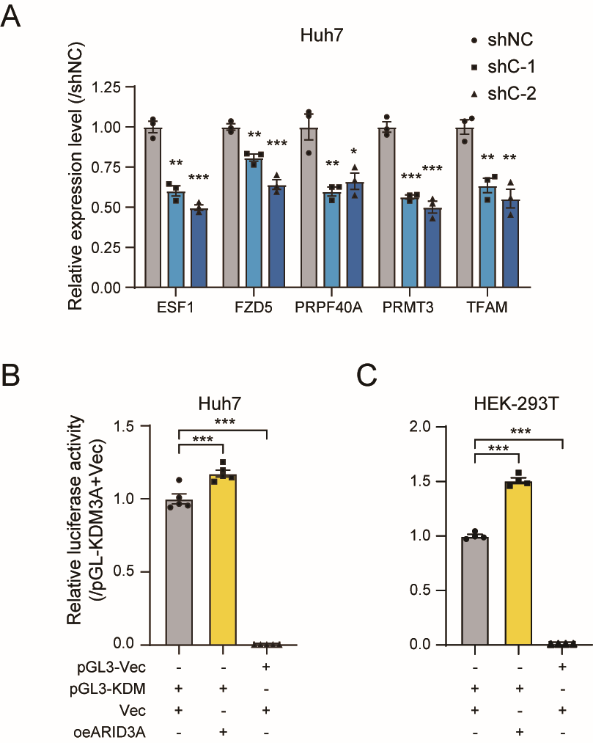


**Fig. S7 ARID3A and CEP131 cooperatively activate KDM3A transcription to regulate liver cancer cell stemness.** **A** The mRNA levels of representative ARID3A-associated ES genes in CEP131-knockdown Huh7 cells. n=3 biologically independent experiments, one-way ANOVA with Tukey’s multiple comparison test. **B and C** Luciferase reporter assay analysis of KDM3A promoter activity in Huh7 **(B)** and HEK-293T **(C)** cells after ARID3A overexpression. n = 5 biologically independent experiments in b, n = 4 biologically independent experiments in c, one-way ANOVA with Tukey’s multiple comparison test. The values are expressed as the means ± SEMs; *p < 0.05, **p < 0.01, and ***p < 0.001.

**Figure S8**


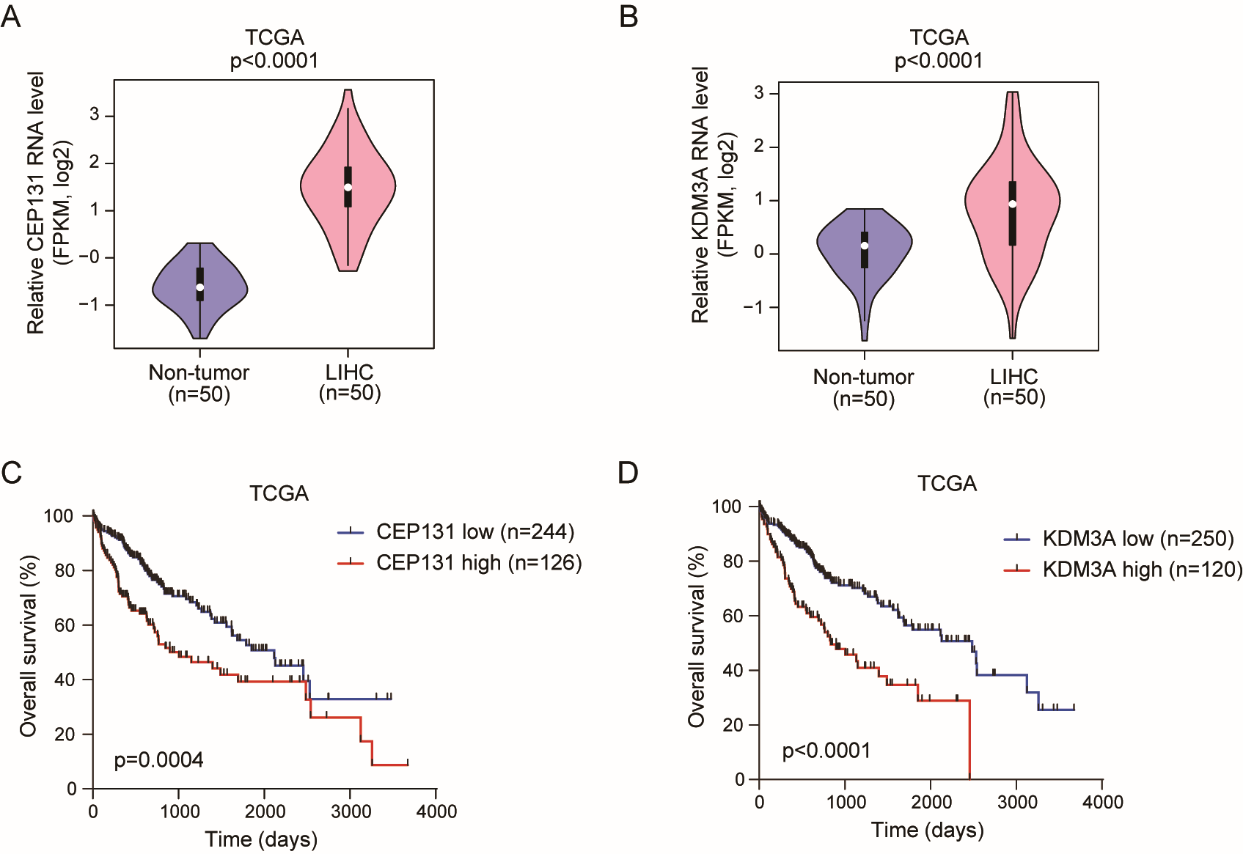


**Fig. S8 CEP131 and KDM3A are upregulated in tumours and high expression of CEP131 or KDM3A correlates with poor outcomes in liver cancer patients. A and B** Expression of CEP131 **(A)** and KDM3A **(B)** were increased in tumour tissues in 50 paired samples in the TCGA-LIHC cohort, two-tailed Student’s *t* test. **C and D** Kaplan–Meier analysis of overall survival for liver cancer patients in the TCGA-LIHC cohort (n = 370), Log-rank test.

**Supplementary** **Tables**

**Table S1. Association between ARID3A expression and clinicopathological variables of TCGA-LIHC patients**

| Clinical Variables | p-value | ARID3A (High) | | ARID3A (Low) | | Total No. | Unknown No. |
| --- | --- | --- | --- | --- | --- | --- | --- |
|  |  | Case No. | % | Case No. | % |  |  |
| Age (yr) | 0.070 |  |  |  |  | 370 | 1 |
| ≥60 |  | 111 | 50.5 | 90 | 60.0 |  |  |
| ＜60 |  | 109 | 49.5 | 60 | 40.0 |  |  |
| Gender | **0.001** |  |  |  |  | 371 | 0 |
| Male |  | 134 | 60.6 | 116 | 77.3 |  |  |
| Female |  | 87 | 39.4 | 34 | 22.7 |  |  |
| TNM Stage | 0.742 |  |  |  |  | 347 | 24 |
| Stage I |  | 101 | 48.6 | 70 | 50.4 |  |  |
| Stage II-IV |  | 107 | 51.4 | 69 | 49.6 |  |  |
| Tumour Grade | **0.001** |  |  |  |  | 366 | 5 |
| G1-G2 |  | 123 | 56.4 | 108 | 73.0 |  |  |
| G3-G4 |  | 95 | 43.6 | 40 | 27.0 |  |  |
| Inflammation | 0.189 |  |  |  |  | 234 | 137 |
| Positive |  | 69 | 53.9 | 48 | 45.3 |  |  |
| Negative |  | 59 | 46.1 | 58 | 54.7 |  |  |
| Vascular Tumour Event | 0.264 |  |  |  |  | 315 | 56 |
| Positive |  | 69 | 37.1 | 40 | 31.0 |  |  |
| Negative |  | 117 | 62.9 | 89 | 69.0 |  |  |
| Child Pugh Classification Grade | 0.634 |  |  |  |  | 239 | 132 |
| A |  | 120 | 91.6 | 97 | 89.8 |  |  |
| B-C |  | 11 | 8.4 | 11 | 10.2 |  |  |
| New Tumour Event | 0.413 |  |  |  |  | 269 | 102 |
| Positive |  | 53 | 33.3 | 42 | 38.2 |  |  |
| Negative |  | 106 | 66.7 | 68 | 61.8 |  |  |

The bold p-values are considered statistically significant

**Table S2. Association between ARID3A expression and clinicopathological variables of 101 primary liver cancer patients**

| Clinical Variables | p-value | ARID3A (High) | | ARID3A (Low) | | Total No. | Unknown No. |
| --- | --- | --- | --- | --- | --- | --- | --- |
|  |  | Case No. | % | Case No. | % |  |  |
| Age (yr) | 0.408 |  |  |  |  | 101 | 0 |
| ≥60 |  | 22 | 28.6 | 9 | 37.5 |  |  |
| ＜60 |  | 55 | 71.4 | 15 | 62.5 |  |  |
| Gender | 0.293 |  |  |  |  | 101 | 0 |
| Male |  | 65 | 84.4 | 18 | 75.0 |  |  |
| Female |  | 12 | 15.6 | 6 | 25.0 |  |  |
| Tumour number | 0.392 |  |  |  |  | 101 | 0 |
| =1 |  | 61 | 79.2 | 17 | 70.8 |  |  |
| ≥2 |  | 16 | 20.8 | 7 | 29.2 |  |  |
| Tumour size | 0.391 |  |  |  |  | 101 | 0 |
| ≥5 |  | 43 | 55.8 | 11 | 45.8 |  |  |
| ＜5 |  | 34 | 44.2 | 13 | 54.2 |  |  |
| Tumour thrombus | **0.035** |  |  |  |  | 101 | 0 |
| Positive |  | 13 | 16.9 | 0 | 0.0 |  |  |
| Negative |  | 64 | 83.1 | 24 | 100.0 |  |  |
| Metastasis | **0.046** |  |  |  |  | 101 | 0 |
| Positive |  | 37 | 48.1 | 6 | 25.0 |  |  |
| Negative |  | 40 | 51.9 | 18 | 75.0 |  |  |
| AFP (μg/L) | 0.770 |  |  |  |  | 92 | 9 |
| ＞25 |  | 33 | 46.5 | 9 | 42.9 |  |  |
| ≤25 |  | 38 | 53.5 | 12 | 57.1 |  |  |
| ALT (U/L) | 0.779 |  |  |  |  | 92 | 9 |
| ＞40 |  | 28 | 39.4 | 9 | 42.9 |  |  |
| ≤40 |  | 43 | 60.6 | 12 | 57.1 |  |  |
| Capsule | 0.213 |  |  |  |  | 99 | 2 |
| Positive |  | 35 | 46.1 | 14 | 60.9 |  |  |
| Negative |  | 41 | 53.9 | 9 | 39.1 |  |  |
| Cirrhosis | 0.694 |  |  |  |  | 100 | 1 |
| Positive |  | 64 | 84.2 | 21 | 87.5 |  |  |
| Negative |  | 12 | 15.8 | 3 | 12.5 |  |  |
| Recurrent | 0.697 |  |  |  |  | 101 | 0 |
| Positive |  | 42 | 54.5 | 12 | 50.0 |  |  |
| Negative |  | 35 | 45.5 | 12 | 50.0 |  |  |

The bold p-values are considered statistically significant

**Table S3. 60 Potential interacting proteins**

| Accession | Gene Name | -10lgP | Unique Peptide(s) | #Spec IP-ARID | #Spec IP-IgG |
| --- | --- | --- | --- | --- | --- |
| Q9UPN4\|CP131_HUMAN | CEP131 | 136.85 | 10 | 10 | 0 |
| Q9UBM7\|DHCR7_HUMAN | DHCR7 | 124.14 | 6 | 7 | 0 |
| Q6KB66\|K2C80_HUMAN | KRT80 | 106.52 | 6 | 6 | 0 |
| P05023\|AT1A1_HUMAN | ATP1A1 | 93.74 | 6 | 6 | 0 |
| P04843\|RPN1_HUMAN | RPN1 | 93.44 | 7 | 9 | 0 |
| P16615\|AT2A2_HUMAN | ATP2A2 | 87.18 | 5 | 5 | 0 |
| Q01469\|FABP5_HUMAN | FABP5 | 81.74 | 3 | 3 | 0 |
| P10809\|CH60_HUMAN | HSPD1 | 80.17 | 3 | 3 | 0 |
| P35321\|SPR1A_HUMAN | SPRR1A | 79.94 | 2 | 2 | 0 |
| P22528\|SPR1B_HUMAN | SPRR1B | 79.94 | 2 | 2 | 0 |
| P36578\|RL4_HUMAN | RPL4 | 77.36 | 2 | 2 | 0 |
| P61619\|S61A1_HUMAN | SEC61A1 | 65.53 | 2 | 2 | 0 |
| P53007\|TXTP_HUMAN | SLC25A1 | 64.46 | 2 | 2 | 0 |
| O75223\|GGCT_HUMAN | GGCT | 61.1 | 2 | 2 | 0 |
| Q8N8E3\|CE112_HUMAN | CEP112 | 58.11 | 2 | 5 | 0 |
| P32119\|PRDX2_HUMAN | PRDX2 | 58.08 | 3 | 3 | 0 |
| P07900\|HS90A_HUMAN | HSP90AA1 | 56.79 | 2 | 2 | 0 |
| Q15758\|AAAT_HUMAN | SLC1A5 | 55.29 | 1 | 1 | 0 |
| P45880\|VDAC2_HUMAN | VDAC2 | 52.2 | 2 | 2 | 0 |
| Q9HCY8\|S10AE_HUMAN | S100A14 | 51.1 | 2 | 2 | 0 |
| P60842\|IF4A1_HUMAN | EIF4A1 | 49.81 | 1 | 1 | 0 |
| P25311\|ZA2G_HUMAN | AZGP1 | 48.26 | 1 | 1 | 0 |
| P06748\|NPM_HUMAN | NPM1 | 47.65 | 2 | 3 | 0 |
| P06576\|ATPB_HUMAN | ATP5F1B | 46 | 1 | 1 | 0 |
| P10599\|THIO_HUMAN | TXN | 45.93 | 1 | 1 | 0 |
| Q8IVW6\|ARI3B_HUMAN | ARID3B | 41.88 | 1 | 2 | 0 |
| P61247\|RS3A_HUMAN | RPS3A | 41.25 | 1 | 2 | 0 |
| P01701\|LV151_HUMAN | IGLV1-51 | 40.42 | 1 | 1 | 0 |
| P01714\|LV319_HUMAN | IGLV3-19 | 40.42 | 1 | 1 | 0 |
| P05090\|APOD_HUMAN | APOD | 40.29 | 1 | 1 | 0 |
| Q86YT6\|MIB1_HUMAN | MIB1 | 40.28 | 3 | 3 | 0 |
| P63244\|RACK1_HUMAN | RACK1 | 39.9 | 1 | 1 | 0 |
| P22735\|TGM1_HUMAN | TGM1 | 39.73 | 1 | 1 | 0 |
| A0A0B4J1V2\|HV226_HUMAN | IGHV2-26 | 39.56 | 1 | 1 | 0 |
| P01814\|HV270_HUMAN | IGHV2-70 | 39.56 | 1 | 1 | 0 |
| A0A0C4DH43\|HV70D_HUMAN | IGHV2-70D | 39.56 | 1 | 1 | 0 |
| P46782\|RS5_HUMAN | RPS5 | 39.23 | 1 | 1 | 0 |
| P62753\|RS6_HUMAN | RPS6 | 38.78 | 1 | 1 | 0 |
| P16520\|GBB3_HUMAN | GNB3 | 37.71 | 1 | 1 | 0 |
| Q9HAV0\|GBB4_HUMAN | GNB4 | 37.71 | 1 | 1 | 0 |
| P62879\|GBB2_HUMAN | GNB2 | 37.71 | 1 | 1 | 0 |
| P62873\|GBB1_HUMAN | GNB1 | 37.71 | 1 | 1 | 0 |
| P35232\|PHB_HUMAN | PHB | 37.31 | 1 | 1 | 0 |
| P14625\|ENPL_HUMAN | HSP90B1 | 37.25 | 3 | 3 | 0 |
| Q58FF3\|ENPLL_HUMAN | HSP90B2P | 37.25 | 1 | 1 | 0 |
| P22695\|QCR2_HUMAN | UQCRC2 | 36.76 | 1 | 1 | 0 |
| Q02878\|RL6_HUMAN | RPL6 | 34.56 | 1 | 1 | 0 |
| Q96L21\|RL10L_HUMAN | RPL10L | 34.55 | 2 | 2 | 0 |
| P27635\|RL10_HUMAN | RPL10 | 34.55 | 2 | 2 | 0 |
| P62826\|RAN_HUMAN | RAN | 33.82 | 1 | 1 | 0 |
| Q14739\|LBR_HUMAN | LBR | 33.63 | 1 | 1 | 0 |
| P49916\|DNLI3_HUMAN | LIG3 | 32.67 | 1 | 1 | 0 |
| P24539\|AT5F1_HUMAN | ATP5PB | 32.26 | 1 | 1 | 0 |
| O75342\|LX12B_HUMAN | ALOX12B | 31.84 | 1 | 1 | 0 |
| P13010\|XRCC5_HUMAN | XRCC5 | 31.77 | 1 | 1 | 0 |
| O15523\|DDX3Y_HUMAN | DDX3Y | 31.72 | 1 | 1 | 0 |
| O00571\|DDX3X_HUMAN | DDX3X | 31.72 | 1 | 1 | 0 |
| Q15738\|NSDHL_HUMAN | NSDHL | 31.32 | 1 | 1 | 0 |
| Q8NHV4\|NEDD1_HUMAN | NEDD1 | 31.29 | 1 | 1 | 0 |
| P08865\|RSSA_HUMAN | RPSA | 30.72 | 1 | 1 | 0 |

**Table S4. Sequences of qPCR primers**

| Identifier | Forward Primer (5'-3') | Reverse Primer (5'-3') |
| --- | --- | --- |
| β-actin | TTGTTACAGGAAGTCCCTTGCC | ATGCTATCACCTCCCCTGTGTG |
| ARID3A | CGACTGGACTTACGAGGAGC | TGAAGCTGAACAAGTCATCCA |
| KDM3A | TTGAGCCACACAGACAGGTT | CACCCAGTGCAGGTTGAAGA |
| CEP131 | GAGACCAGAGTGCCAGGAAT | CTGTGGTTCTGGGGATGAGT |
| BMPR1A | ACCTGGGCCTTGCTGTTAAA | TGTCAGCCATGATGTAGGGC |
| JAG1 | ACCCCCTGTGAAGTGATTGAC | ACTGACTCTTGCACTTCCCG |
| SMC3 | CAGACAACCGGTTACCAATCG | AGCGCTTTCAAGGAGGTTCA |
| TBX3 | CCAAGTCGGGAAGGCGAAT | AACGACAGTCATCAGCAGCTA |
| WDR43 | ACGCCAGTTTCGTCACTGAT | GATCGGACCTGCCAGACATT |
| Chr-KDM3A | GATAACCGATTTCGCGGGAC | CGAGTGATCCCTCCGCCATA |
| Chr-BMPR1A | CCAGCAATAGCCGTGTGAGA | ACTGACAAGCAGGCCAAAGA |
| Chr-JAG1 | TATTCATGAGAGGGCGTGCT | AAGTTCCTCCTCGCACTACC |
| Chr-SMC3 | TTAAACAGCGCGGAATCACG | ACAAAGGGGGAAAGGTTCCG |
| Chr-TBX3 | TCTTTGACGCTTTCGGACCA | TCTATCCCCCAGCACTCGAC |
| Chr-WDR43 | CCCGCCCAGAACAAGATAGC | CTCAGGGAGTGGATCTTCGC |
| ESF1 | GAGGATTGTGATGGCCTGGA | TCCCAAGTGATTTCCACCGTT |
| FZD5 | GGGATCCGTGGAGAGTCCTT | GGCAACCTGTTGGTTGCTTT |
| PRMT3 | GGAGGATGAGGACGATGCAG | TCAGCAGATGTGAATAACCTGT |
| PRPF40A | CCAATGGGACAAATGCCTGG | TTTACTCCTGGCGGTAAGGC |
| TIA1 | TGCCCAAGACTCTATACGTCG | AGGGGTTGTTGCCCAATTCA |
| Chr-ESF1 | GCACGTTCCCTCGAGAAGAT | AATTGCCGGCGCTTTTTGTG |
| Chr-FZD5 | CGCTCCAGTGGACTCCTG | GAGGTGGGCGAAAGGGTG |
| Chr-PRMT3 | TGAAGGAGGTGCTGAGTGAG | TCTGCATCGTCCTCATCCTC |
| Chr-PRPF40A | GGAGGATGGCTCTCCATTTCA | GTTGGTATGTGTAGCGGCAG |
| Chr-TIA1 | TCTTGCATGATACCACTACACC | TCCTAAAATACTGTGGCGGC |
| myco-qPCR1 | GTGCTGGATATCCCGGGCTAAGC | AGGCGAACCGTTCACTCCCC |
| myco-qPCR2 | GGGAGCAAACAGGATTAGTATCCCT | TGCACCATCTGTCACTCTGTTAACCTC |
| myco-qPCR3 | ACGCGTAGAACCTTACCCAC | ACGACAACCATGCACCATCT |

**Table S5. Sequences of siRNAs used in this study**

| Identifier | Sequences (5'-3') |
| --- | --- |
| siARID3A-1 | ACATCTACCTCAAATAACT |
| siARID3A-2 | CTTACGAGGAGCAGTTTAA |
| siKDM3A-1 | GCATTTAGATGAAAGCCAT |
| siKDM3A-2 | GCTCCACATCAGGTTCATA |
| siCEP131-1 | CAGCCATTCAGGAGCTGCAACAGAA |
| siCEP131-2 | GAGGCCAAGCTACAAAGCATCATGA |

**Table S6. Sequences of primers used in this study**

| Identifier | Sequences (5'-3') |
| --- | --- |
| ARID3A_F | CCTCGAGGTTTAAACTACGGATGAAACTACAGGCCGTGATGGAG |
| ARID3A_R | ATCATATGACTAGTCCCGGGTTAAGGCAACGAGTTATTTGAGGTA |
| FLAG-ARID3A-FL_F | CAGTCGACTGGAATGAAACTACAGGCCGTGAT |
| FLAG-ARID3A-FL_R | CGCGGCCGCGGATTAAGGCAACGAGTTATTTG |
| FLAG-ARID3A-ab_F | ATGACGATGACAAGGAATTCATGAAACTACAGGCCGTGAT |
| FLAG-ARID3A-ab_R | TCGCTGCAGCTCGAGCCCGGGTCACTCTTTCTTGATCTTAGGGG |
| FLAG-ARID3A-c_F | ATGACGATGACAAGGAATTCAGCAACCGACGGGAGGGCCG |
| FLAG-ARID3A-c_R | TCGCTGCAGCTCGAGCCCGGGTCAAGGCAACGAGTTATTTG |
| shARID3A_F1 | CACCGGGACTTACGAGGAGCAGTTTCAAGAACTGCTCCTCGTAAGTCCTTTTTT |
| shARID3A_R1 | AAACAAAAAAGGACTTACGAGGAGCAGTTCTTGAAACTGCTCCTCGTAAGTCCC |
| shARID3A_F2 | CACCGCTTACGAGGAGCAGTTTAATCAAGTTAAACTGCTCCTCGTAAGTTTTTT |
| shARID3A_R2 | AAACAAAAAACTTACGAGGAGCAGTTTAACTTGATTAAACTGCTCCTCGTAAGC |
| promoter-ARID3A_F | CTCTATCGATAGGTACCGAGCGGGTTTGGAGCTGGGAGAAG |
| promoter-ARID3A_R | AGATCGCAGATCTCGAGCCAACGAACACCGTGGTGCTGG |
| enhancer-ARID3A_F | CTCTATCGATAGGTACCGAGCGGGTTTGGAGCTGGGAGAAG |
| enhancer-ARID3A_R | AGATCGCAGATCTCGAGCCAACGAACACCGTGGTGCTGG |
| HA-CEP131-FL_F | CGATGTTCCAGATTACGCTATGAAAGGCACCCGGGCCAT |
| HA-CEP131-FL_R | CAGATCCTTCGCGGCCGCTCACTTGGTACTTGGCGTGG |
| HA-CEP131-a_F | ATGACGTTCCAGATTACGCTATGAAAGGCACCCGGGCCAT |
| HA-CEP131-a_R | CGCGGATCCGATTTAAATTCTCATCTCAGGGCTCGTTTCTGTT |
| HA-CEP131-b_F | ATGACGTTCCAGATTACGCTCGGGAGGAGCAGCGGCAGCG |
| HA-CEP131-b_R | CGCGGATCCGATTTAAATTCTCAGCGCAGGCAGCGCTGCGAGG |
| HA-CEP131-cd_F | ATGACGTTCCAGATTACGCTAAGAAGATCAAGGAGGTCAC |
| HA-CEP131-cd_R | CGCGGATCCGATTTAAATTCTCACTTCTCACTCTCCTCCTTGG |
| HA-CEP131-de_F | ATGACGTTCCAGATTACGCTCACCAGATGGAGCTGAATAC |
| HA-CEP131-de_R | CGCGGATCCGATTTAAATTCTCACTTGGTACTTGGCGTGGGCC |
| shCEP131_F1 | CACCGTCTCTGACAGCTGACAACTTGGAGATCAAGTCTCCAAGTTGTCAGCTGTCAGAGATTTTTT |
| shCEP131_R1 | AAACAAAAAATCTCTGACAGCTGACAACTTGGAGACTTGATCTCCAAGTTGTCAGCTGTCAGAGAC |
| shCEP131_F2 | CACCGCCATCAACAACCTTAGAAGATCAAGTCTTCTAAGGTTGTTGATGGCTTTTTT |
| shCEP131_R2 | AAACAAAAAAGCCATCAACAACCTTAGAAGACTTGATCTTCTAAGGTTGTTGATGGC |
| HA-KDM3A_F | CGATGTTCCAGATTACGCTATGGTGCTCACGCTCGGA |
| HA-KDM3A_R | CAGATCCTTCGCGGCCGCTTAAGGTTTGCCAAAACTGGA |
| enhancer-KDM3A_F | ATTTCTCTATCGATAGGTACTCCAGAGCACACAATCACCT |
| enhancer-KDM3A_R | GCTTACTTAGATCGCAGATCCTCTCACAAGACACGCACAG |
| shKDM3A_F1 | CACCGCATTTAGATGAAAGCCATCTTCAAGAGATGGCTTTCATCTAAATGCTTTTTT |
| shKDM3A_R1 | AAACAAAAAAGCATTTAGATGAAAGCCATCTCTTGAAGATGGCTTTCATCTAAATGC |
| shKDM3A_F2 | CACCGGTGCTGCTTACAAGACTTTCTCAAGGAAAGTCTTGTAAGCAGCACCTTTTTT |
| shKDM3A_R2 | AAACAAAAAAGGTGCTGCTTACAAGACTTTCCTTGAGAAAGTCTTGTAAGCAGCACC |

**Table S7. Antibodies used in this study**

| Identifier | Catalog Number | Company |
| --- | --- | --- |
| GAPDH | 60004-1-Ig | Proteintech |
| ARID3A | sc-398367 | Santa Cruz |
| CEP131 (AZI1) | ab99379 | Abcam |
| KDM3A | 12835-1-AP | Proteintech |
| Goat anti-Mouse IgG (H+L), HRP conjugate | SA00001-1 | Proteintech |
| Goat anti-Rabbit IgG (H+L), HRP conjugate | SA00001-2 | Proteintech |
| Flag Tag | F1804 | Sigma-aldrich |
| HA Tag | 51064-2-AP | Proteintech |
| IgG | I5381 | Sigma-aldrich |
| APC anti-human CD326 (EpCAM) | 324207 | Biolegend |
| PE anti-human CD13 | 301703 | Biolegend |
