# Supplementary figures and images for: Hepatic ARID3A facilitates liver cancer malignancy by cooperating with CEP131 to regulate an embryonic stem cell-like gene signature
Source: Cell Death Dis. 2022 Aug 25;13(8):732. doi: 10.1038/s41419-022-05187-9 (PMC9411159; doi:10.1038/s41419-022-05187-9)

Fig. 4e

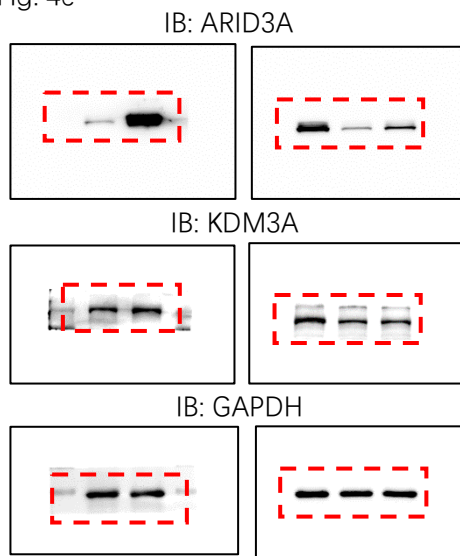

Fig. 5c

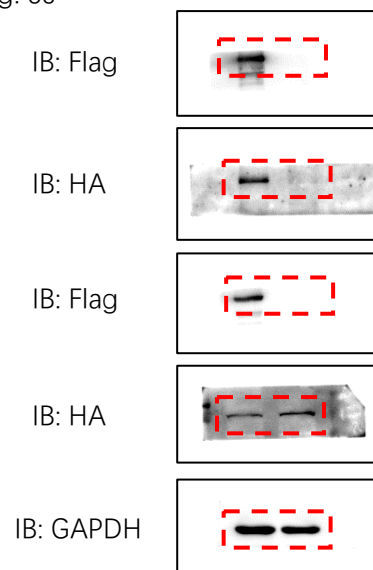

Fig. 5d

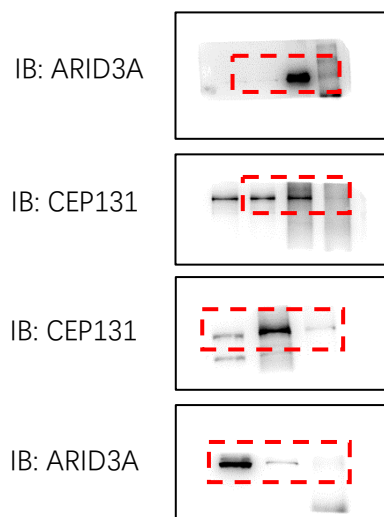

Fig. 5e

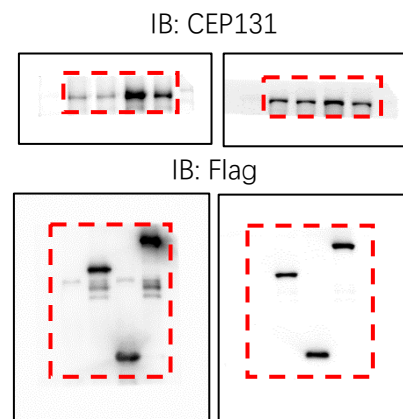

Fig. 5f

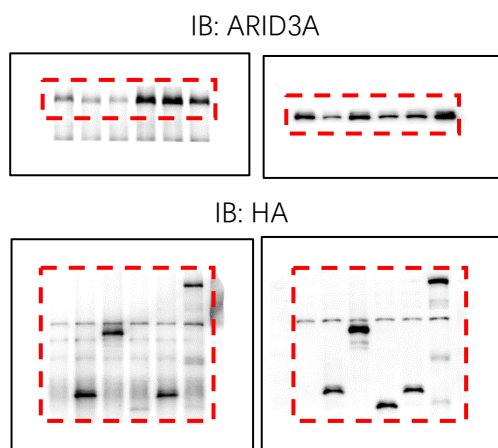

Fig. 6b

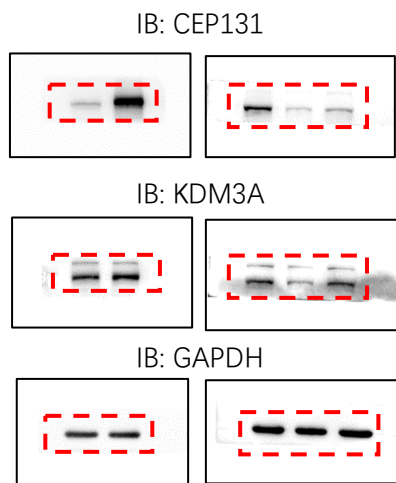

Fig. S2b

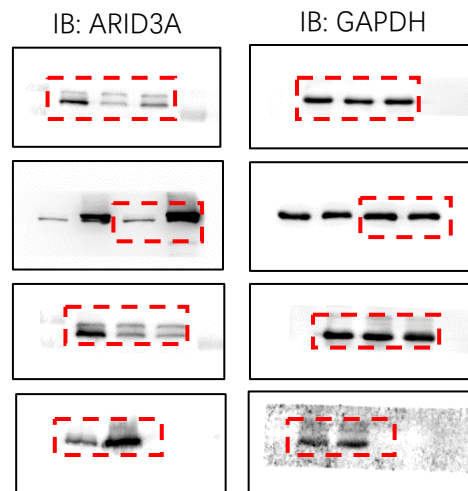

Fig. S4h

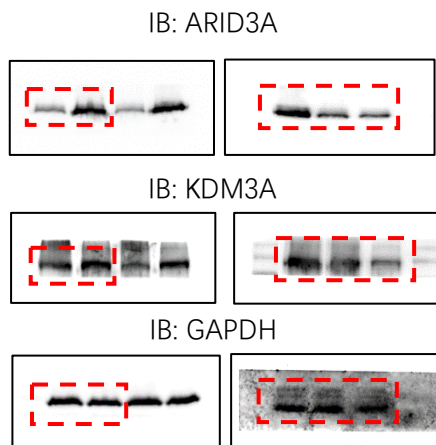

Fig. S6a

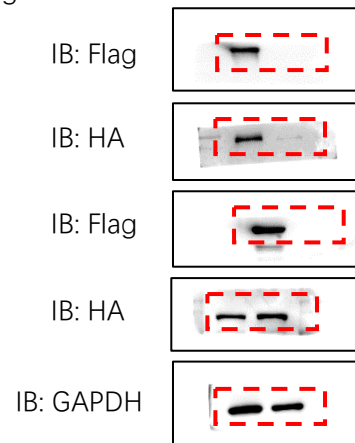

Fig. S6b

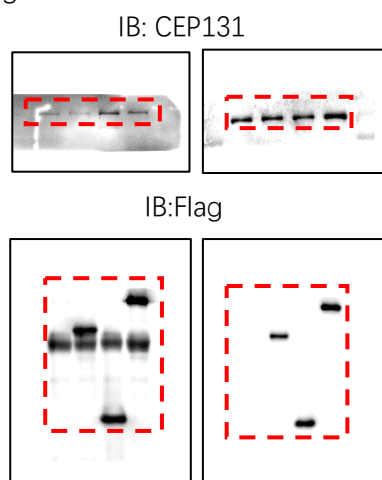

Fig. S6c

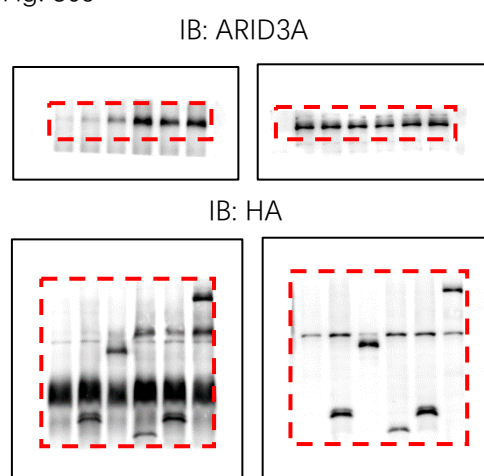

Supplement: Supplementary file 3 — Full length upcropped original western blots [file 41419_2022_5187_MOESM3_ESM.pdf]
